# Lightweight, Elastic Ceramic Fabrics for Broadband Electromagnetic Absorption and High Temperature Thermal Insulation

**DOI:** 10.1002/advs.76453

**Published:** 2026-07-13

**Authors:** Jiahao Yang, Qi Ding, Juanjuan Xu, Fei Yan, Rui Wang, Yuena Zhang, Chao Zhao, Zhi Cheng, Yuchi Fan, Wan Jiang

**Affiliations:** ^1^ State Key Laboratory of Advanced Fiber Materials, College of Materials Science and Engineering Donghua University Shanghai China

**Keywords:** elastic ceramic nanofiber fabrics, electromagnetic wave absorption, high temperature thermal insulation, SiBCNZr, ultrafast high‐temperature sintering

## Abstract

With the rapid advancement of communication and radar detection in aerospace applications, developing a high‐performance electromagnetic wave (EMW) absorber with mechanical elasticity and high‐temperature thermal insulation remains urgent and challenging. Here, lightweight, elastic SiBCNZr ceramic nanofiber fabrics with outstanding electromagnetic absorption (EMA) and high‐temperature thermal insulation were developed by precisely controlling the microstructure during ultrafast high‐temperature sintering (UHS). The fabrics achieved a minimum reflection loss (*RL*_min_) of −83.33 dB and an effective absorption bandwidth (EAB) of 9.8 GHz, covering the entire X‐ and Ku‐bands. This performance arose from a balance between impedance matching and EMW attenuation, enabled by UHS‐controlled precipitation of defect‐rich *t*‐ZrO_2_ nanograins and turbostratic C nanoclusters. Furthermore, the fabrics showed elastic resilience up to 60% compressive strain, attributed to defect‐assisted dislocation activity, interfacial accommodation, nanoscale fiber diameters, and weak inter‐fiber friction. Finally, the fabrics exhibited excellent thermal insulation (∆T ≈ 900°C) and low thermal conductivity (0.0603 W·m^−1^·K^−1^), because heat transport in both the solid and gas phases was suppressed by nanoscale grains, grain defects, abundant heterointerfaces, and high porosity. This work establishes an effective strategy for simultaneously enhancing EMA, mechanical elasticity, and thermal insulation, highlighting the potential of these fabrics for advanced aerospace applications.

## Graphical abstract

This study reports lightweight, elastic ceramic fabrics with broadband electromagnetic absorption (EAB = 9.8 GHz) and high‐temperature thermal insulation, enabled by precise microstructural control during ultrafast high‐temperature sintering (UHS), which induces the precipitation of defect‐rich *t*‐ZrO_2_ nanograins and turbostratic C nanoclusters. This work provides design guidelines for high‐performance ceramic fabrics for electromagnetic absorption and thermal protection in aerospace applications.

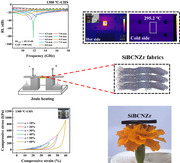

## 1 Introduction

With the rapid development of communication and radar‐detection technologies, developing high‐performance electromagnetic wave (EMW) absorbers that combine mechanical elasticity with high‐temperature thermal insulation is urgently needed, yet remains challenging for radar stealth and thermal protection in aerospace applications [1, 2, 3, 4]. High‐performance EMW absorbers require strong absorption, broad bandwidth, low density, and thin thickness, which in turn demands a balance between EMW attenuation and impedance matching [5, 6]. A typical strategy is to combine an EMW‐transparent matrix with EMW attenuation components that provide conductive, dielectric, and/or magnetic losses, such as MXenes [7, 8, 9], graphene [10, 11, 12], carbon nanotubes [13, 14], (metal–organtic frameworks) MOFs [15, 16, 17], and magnetic materials [18, 19, 20]. Recent efforts focus on microstructural design, such as geometry [21], defects [22], heterointerfaces [23], high‐entropy compositions [24], and built‐in electric fields [25], to improve EMW absorption. However, many reported EMW absorbers show limited high‐temperature stability and poor oxidation resistance, which hinder their operation in harsh thermal environments [26, 27]. Therefore, developing EMW absorbers that combine high‐temperature stability with oxidation resistance remains a key challenge [28].

Polymer‐derived ceramics (PDCs) are well suited for extreme high‐temperature environments because of their exceptional thermal stability and oxidation resistance [29, 30, 31]. The physical and chemical properties of PDCs can be tailored through rational design of the polymer precursors [32]. Among PDCs, SiBCN ceramics remain stable above 1600°C, making them promising candidates for high‐temperature components [33, 34, 35]. Moreover, their electrical properties and electromagnetic absorption (EMA) performance can be tuned through microstructural design [36]. However, their inherent brittleness compromises reliability and durability in practical applications [37]. SiBCNZr ceramic fibers offer excellent mechanical flexibility, which helps mitigate sudden catastrophic fracture during service [38]. However, the slow heating rates in conventional high‐temperature annealing can induce excessive grain growth and graphitization, hindering precise microstructural control and the balance between electromagnetic attenuation and impedance matching, thereby limiting broadband EMA performance [39, 40].

Ultrafast high‐temperature sintering (UHS) offers several advantages, including suppressing grain growth, preventing pore coalescence, mitigating element volatilization, and minimizing interdiffusion. These benefits arise from its rapid heating and cooling rates (10^3^°C–10^4^°C·min^−1^), which facilitate the fabrication of high‐performance ceramics [41]. For instance, a solid‐state electrolyte, Li_6.5_Nd_3_Zr_1.5_Ta_0.5_O_12_, fabricated by UHS achieved a high ionic conductivity of 2.3 × 10^−4^ S·cm^−1^, attributed to a dense microstructure, and low impurity content [42]. Similarly, UHS‐processed BaTiO_3_ ceramics exhibit a high dielectric loss [43]. Collectively, these results suggest that UHS enables precise control over microstructure and electrical/dielectric responses, offering a route to decouple electromagnetic attention from impedance matching and thereby achieve strong, broadband EMA.

Herein, the microstructure and EMA performance of the SiBCNZr fabrics were precisely modulated by UHS at 1100°C–1500°C, accompanied by the precipitation of defect‐rich tetragonal zirconia (*t*‐ZrO_2_) nanograins and turbostratic C nanoclusters from the amorphous fibrous matrix. As a result, the fabrics after UHS at 1300°C exhibited strong, broadband EMW absorption, delivering a minimum reflection loss (*RL*_min_) of −83.3 dB and an effective absorption bandwidth (EAB) of 9.8 GHz. This is primarily attributed to the precipitation of moderate EMW loss phases (*t*‐ZrO_2_ nanograins and turbostratic C) from the EMW‐transparent fibrous matrix, which balances EMW attenuation and impedance matching. Moreover, the fabric has a low density (0.109 g·cm^−3^) and shows excellent elastic resilience under compression up to 60% strain. The fabric also exhibits a low thermal conductivity of 0.0602 W·m^−1^·K^−1^ and excellent high‐temperature thermal insulation with a temperature gradient of 180°C·mm^−1^. This work provides an effective strategy for developing lightweight high‐performance EMW absorbers that integrate low density, mechanical elasticity, and high‐temperature thermal insulation, showing their potential for operation in extreme environments, including aerospace.

## 2 Results and Discussion

**FIGURE 1:**
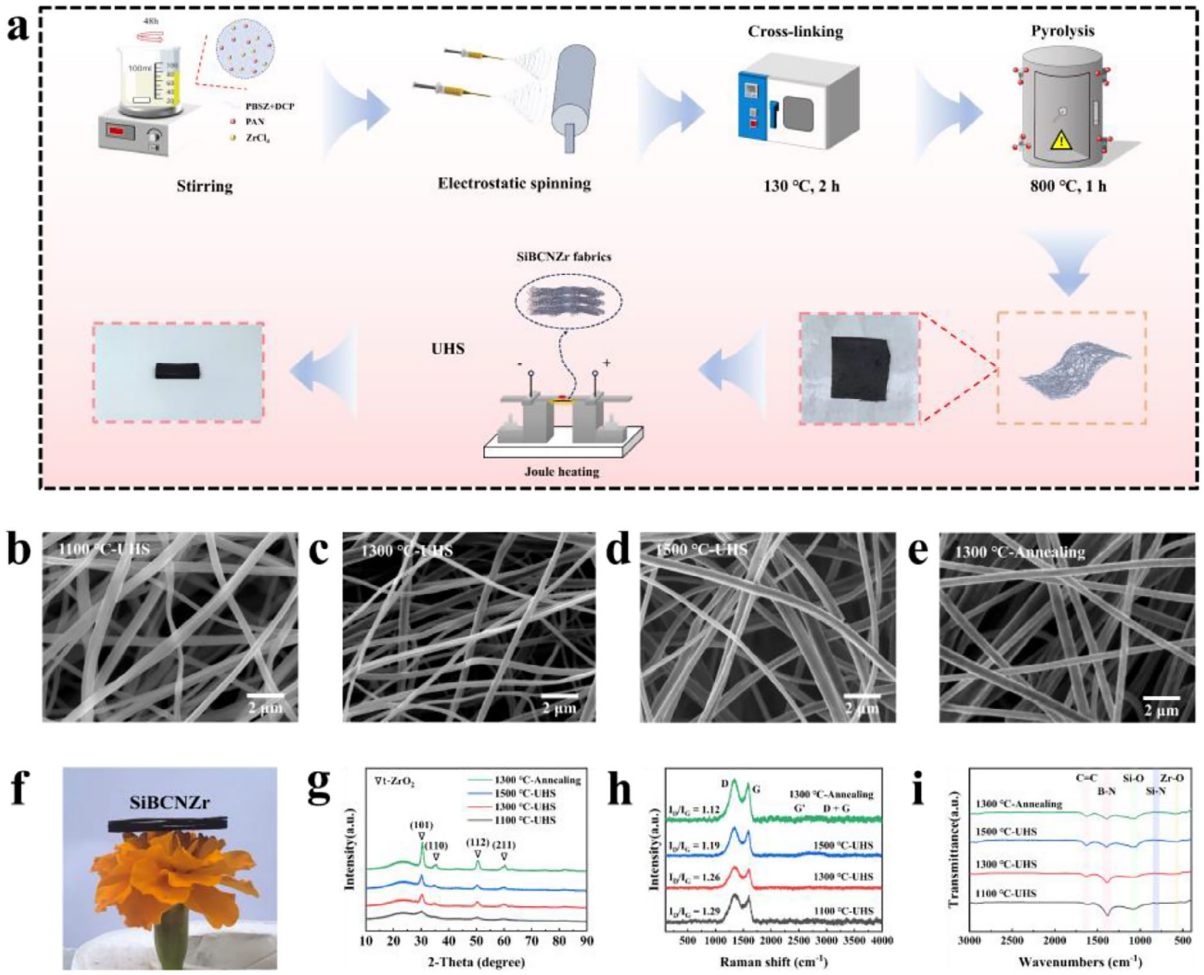
Fabrication and characterization of the SiBCNZr ceramic nanofiber fabrics. (a) Schematic illustration of the fabrication process for the fabrics, including electrostatic spinning, cross‐linking, pyrolysis, and ultrafast high‐temperature sintering (UHS). (b–e) SEM images of the fabrics after UHS at 1100, 1300, 1500°C, and after conventional annealing at 1300°C. (f) Optical photograph showing the fabrics placed on a flower. (g–i) XRD patterns, Raman spectra, and FTIR spectra of the fabrics after UHS and conventional annealing.

SiBCNZr ceramic nanofiber fabrics were fabricated by electrostatic spinning, cross‐linking, pyrolysis, and UHS at 1100°C–1500°C (Figure 1a). As a control, the pyrolyzed fabrics were subjected to conventional annealing at 1300°C. The morphology of SiBCNZr fibers before and after UHS/annealing was observed by SEM (Figure 1b–e; Figure S1). The fabrics pyrolyzed at 800°C had fiber diameters of 100–700 nm and exhibited smooth surfaces (Figure S2a). After UHS or annealing, these fibers retained their morphology and smooth surfaces (Figures S2b–e). Moreover, the fabrics exhibit similarly low densities across the different heat‐treatment conditions. For instance, the density of the fabrics after UHS at 1300°C is as low as 0.109 g·cm^−3^. When the fabrics were placed on a flower, no visible deformation was observed (Figure 1f), further supporting their low density. In short, lightweight SiBCNZr ceramic nanofiber fabrics were obtained based on the UHS method.

The phase composition and grain size of the SiBCNZr fabrics were characterized by XRD (Figure 1g and Figure S3). The fabrics pyrolyzed at 800°C showed no distinct diffraction peaks, indicating an amorphous structure. After UHS at 1100°C, diffraction peaks at 30.2°, 35.3°, 50.2°, and 60.2° were observed, corresponding to the (101), (110), (112), and (211) planes of *t*‐ZrO_2_ [44]. With increasing UHS temperature, the full width at half maximum (FWHM) of the diffraction peaks gradually decreased, indicating grain growth of *t*‐ZrO_2_ [45]. As the UHS temperature increase to 1100°C, 1300°C, and 1500°C, the grain size of *t*‐ZrO_2_ increased to 3.65, 6.41, and 6.78 nm, respectively. In contrast, the fabrics annealed at 1300°C exhibited a grain size of 7.90 nm, larger than that obtained by UHS at 1300°C. In short, UHS offers a clear advantage for precisely tuning the grain size of *t*‐ZrO_2_.

Raman spectroscopy was employed to evaluate the graphitization degree of the SiBCNZr fabrics (Figure 1h and Figure S4, Supporting Information). The D band at ∼1340 cm^−^^1^ is associated with disordered carbon and is commonly attributed to sp^3^‐hybridized carbon, whereas the G band at ∼1590 cm^−1^ corresponds to graphitic carbon and is associated with the sp^2^‐hybridized carbon. A lower *I*_D_/*I*_G_ ratio indicates a higher graphitization degree [46, 47]. With increasing UHS temperature, *I*_D_/*I*_G_ decreased from 1.31 to 1.19, indicating progressive graphitization. At 1500°C, the emergence of the G' and D+G bands suggests further graphitic ordering, which is expected to increase conductive loss. Notably, the SiBCNZr fabric annealed at 1300°C exhibited a lower *I*_D_/*I*_G_ of 1.12 and showed G' and D+G bands, indicating a higher graphitization degree than that of the fabrics after UHS at 1300°C. In short, the graphitization degree of the SiBCNZr fabrics were can be tuned by the UHS process.

The chemical structure of the fabrics was further characterized by Fourier‐transform infrared (FTIR) spectroscopy (Figure 1i and Figure S5). The peaks at 1600, 820, and 1070 cm^−1^ are assigned to the stretching vibration of C = C, the vibration of Si─N, and the stretching vibration of Si─O, respectively [48, 49, 50]. As the UHS temperature increases, the intensity of the B─N peak at 1380 cm^−1^ gradually decreases [51], implying a progressive enhanced electrical conductivity of the fabrics. Furthermore, the intensity of the Zr─O peak at 560 cm^−1^ steadily increases, suggesting the formation of ZrO_2_ within the SiBCNZr fabrics, consistent with the XRD results.

**FIGURE 2:**
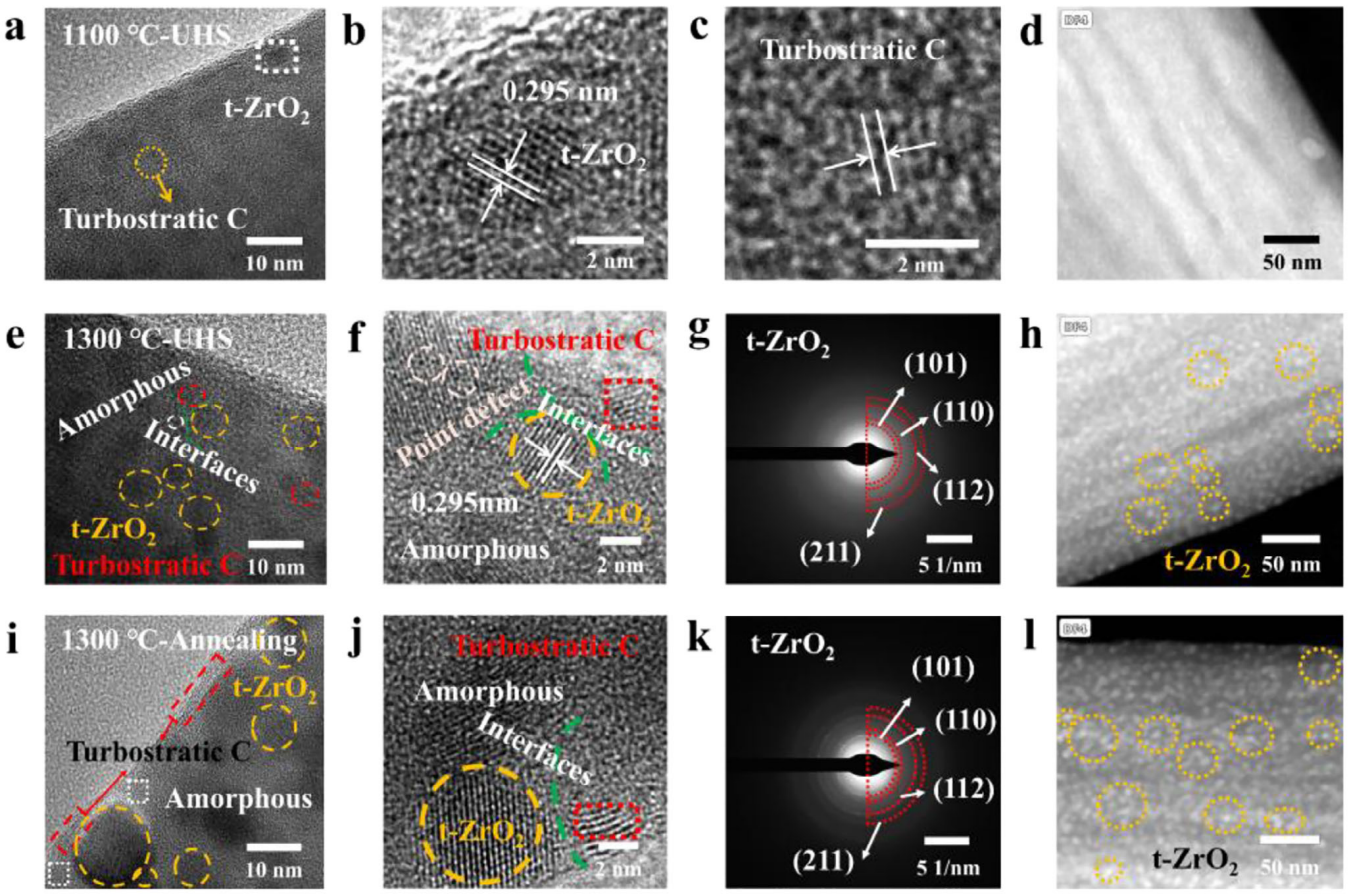
Microstructure of the SiBCNZr fabrics. (a–c) High‐resolution transmission electron microscopy (HRTEM) images, and (d) scanning transmission electron microscopy (STEM) image of the fabrics after UHS at 1100°C; (e, f) HRTEM image, (g) selected‐area electron diffraction (SAED) pattern, and (h) STEM image of the fabrics after UHS at 1300°C; (i, j) HRTEM images, (k) SAED pattern, and (l) STEM image of the fabrics after conventional annealing at 1300°C.

To gain further insight into the microstructure of the SiBCNZr fiber fabrics before and after UHS or annealing, transmission electron microscopy (TEM) was performed (Figure 2 and Figures S6 and S7). For the fabrics pyrolyzed at 800°C, no distinct diffraction spots were observed in the selected‐area electron diffraction (SAED) pattern (Figure S6), indicating an amorphous structure, consistent with the XRD results (Figure S3). After UHS at 1100°C, nanograins with an average diameter of 3.74 nm were observed in the HRTEM images and STEM image (Figure 2a,b,d). Lattice fringes with an interplanar spacing of 0.295 nm were assigned to the (101) plane of *t*‐ZrO_2_ [52]. In addition, turbostratic C nanoclusters were observed in the HRTEM image (Figure 2a,c). After UHS at 1300°C, the *t*‐ZrO_2_ nanograins with an average size of 6.06 nm and a lattice spacing of 0.295 nm were observed (Figure 2e–h). Point defects were observed within these *t*‐ZrO_2_ nanograins (Figure 2f). In addition, turbostratic C nanoclusters with an interplanar spacing of 0.33 nm were identified (Figure 2e,f) [53]. Abundant heterointerfaces formed among these *t*‐ZrO_2_ nanograins, turbostratic C nanoclusters, and the amorphous fibrous matrix (Figure 2f). After UHS at 1500°C, the grain size of defect‐rich *t*‐ZrO_2_ nanograins increase to 6.6 nm, and additional turbostratic C nanoclusters were formed (Figures S7 and S8). In contrast, for the fabrics after annealing at 1300°C, the *t*‐ZrO_2_ nanograins further coarsened, and turbostratic C nanoclusters showed higher graphitic ordering (Figure 2i–l). Overall, with increasing UHS temperature, the defect‐rich *t*‐ZrO_2_ nanocrystals grew progressively and the ordering of the turbostratic C increased, and more heterointerfaces formed. This evolution can increase the carrier and dipole densities, which are expected to enhance conduction and polarization losses under EMW.

**FIGURE 3:**
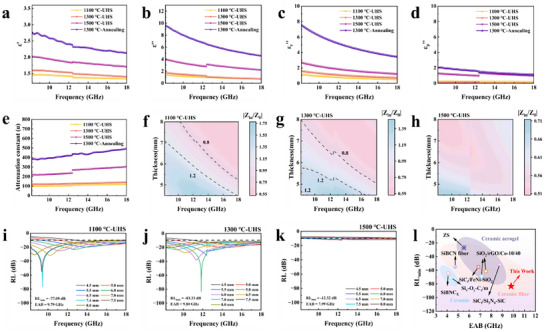
Electromagnetic parameters and EMA performance of the fabrics after UHS/annealing. (a) Real permittivity (*εʹ*), (b) imaginary permittivity (*εʹʹ*), (c) conductive loss (*ε_c_ʹʹ*), (d) polarization loss (*ε_p_ʹʹ*), and (e) attenuation coefficient of the fabrics after UHS/annealing. |*Z_in_*/*Z_0_* | values of the fabrics after UHS at (f) 1100°C, (g) 1300°C, and (h) 1500°C. Reflection loss (*RL*) values of the fabrics after UHS at (i) 1100°C, (j) 1300°C, (k) 1500°C. (l) Comparison of *RL*_min_ and EAB with representative EMW absorbers reported in the literature.

To evaluate the EMA performance of the SiBCNZr fiber fabrics in the X‐ and Ku‐bands (8.2–18 GHz), the relative complex permittivity (ε_*r*_ = ε′  − *j*ε′′) and relative complex permeability (μ_*r*_ = μ′  − *j*μ′′) were measured. Because the fabrics are non‐magnetic, *μ′* remains close to 1 and *μ″* is near 0; therefore, the contribution of permeability to EMA is negligible. *ε′* represents the ability to store electromagnetic energy, while *ε″* reflects the ability to dissipate electromagnetic energy. For all the SiBCNZr fabrics, *ε′* and *ε″* decreased with increasing frequency, showing pronounced frequency dispersion (Figure 3a,b and Figure S9). This behavior can be described by the Debye relaxation model [54, 55]:

(1) $$\varepsilon^{\prime }=\varepsilon_{\infty }+\frac{\varepsilon_{s}-\varepsilon_{\infty }}{1+{\left(\omega \tau \right)}^{2}}$$

(2) $$\varepsilon^{\prime \prime }=\frac{\left(\varepsilon_{s}-\varepsilon_{\infty }\right)\omega \tau }{1+{\left(\omega \tau \right)}^{2}}\;$$

here, *ɛ_∞_* and *ɛ_s_* are the optical and static dielectric constants, respectively; *ω* is the angular velocity (*ω =* 2*πf*, where *f* is frequency), and *τ* is the relaxation time. According to Equations (1) and (2), *ε′* and *ε″* gradually decrease with the increasing frequency, because dipolar polarization cannot fully follow rapidly alternating electric fields at high frequencies. Moreover, the slight discontinuity observed at 12.4 GHz falls within acceptable experimental variation and is associated with the independent waveguide measurements performed in the X‐ and Ku‐bands.

For the fabrics pyrolyzed at 800°C, *ε′* and *ε″* were close to 1 and 0, respectively (Figure S9a), indicating limited EMW attenuation capability. With increasing UHS temperature, *ε′* and *ε″* of SiBCNZr fabrics gradually increased (Figure 3a,b). At 1300°C, *ɛ′* ranged from 1.4 to 1.6, and *ɛ″* ranged from 0.7 to 1.7. In contrast, for fabrics after annealing at 1300°C, *ɛ′* remained 2.1–2.8, and *ɛ″* increased to 4.6–9.6. The dielectric loss tangent ($\tan\;\delta =\frac{\varepsilon^{\prime \prime }}{\varepsilon^{\prime }}$), defined as the ratio of dissipated to stored energy, reflects dielectric relaxation and polarization processes. With increasing UHS temperature, the tan *δ* of fabrics gradually increased (Figure S9b), consistent with the trend in *ε″*.

According to Debye theory, *ɛ″* can be decomposed into conduction loss (*ɛ_c_′′*) and polarization loss (*ɛ_p_′′*), as expressed by the following equations [56]:

(3)

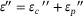



(4)

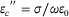

here, *ɛ_c_′′* and *ɛ_p_′′* represent conduction and polarization losses, respectively; *σ* is the electrical conductivity; *ɛ_0_* is the vacuum permittivity (8.85×10^−12^ F/m); and *ω* is the angular frequency (*ω* = 2*πf*, where *f* is the electromagnetic wave frequency). Because *ɛ_c_′′* is strongly correlated with the *σ*, the conductivity of the fabrics was measured. With increasing UHS temperature, *σ* of the fabrics gradually increased (Figure S10). After UHS at 1300°C, the *σ* of fabrics increases to 0.732 S·m^−1^. In contrast, *σ* for the fabrics annealed at 1300°C increased to 3.44 S·m^−1^. According to Equation (4), *ɛ_c_′′* was calculated for each fabric. The *ɛ_c_′′* values of the fabrics exhibited a similar increasing trend (Figure 3c). The *ɛ_p_′′* values of the fabrics were further calculated according to Equation (3) (Figure 3d and Figure S11). With increasing UHS temperature, the *ɛ_p_′′* of the fabrics also increased. For all the fabrics after UHS, *ɛ_c_′′* was larger than *ɛ_p_′′*, indicating that conductive loss (*ɛ_c_′′*) plays a dominant role in EMW attenuation. In addition, the Cole–Cole plots of all fabrics are shown in Figure S12. For the raw fabrics pyrolyzed at 800°C and those after UHS, several semicircles were observed, representing multiple polarization relaxations. These features can be attributed the dipolar polarization deriving from *t*‐ZrO_2_ nanograins and turbostratic C nanoclusters, as well as interfacial polarization at heterointerfaces among the *t*‐ZrO_2_ nanograins, turbostratic C nanoclusters, and amorphous fibrous matrix.

The attenuation coefficient (*α*) is a key parameter for EMA and is determined by the real and imaginary parts of the complex permittivity and permeability. *α* was calculated using the following equation [57, 58, 59]:

(5) $$\alpha =\frac{\sqrt{2}}{c}\pi f\sqrt{\left(\mu^{\prime \prime }\varepsilon^{\prime \prime }-\mu^{\prime }\varepsilon^{\prime }\right)+\sqrt{{\left(\mu^{\prime \prime }\varepsilon^{\prime \prime }-\mu^{\prime }\varepsilon^{\prime }\right)}^{2}+{\left(\mu^{\prime }\varepsilon^{\prime \prime }+\mu^{\prime \prime }\varepsilon^{\prime }\right)}^{2}}}$$

For the fabrics pyrolyzed at 800°C, *α* ranged from 20 to 25 (Figure S13). With increasing UHS temperature, *α* increased, indicating stronger EMW attenuation (Figure 3e). When the UHS temperature reached 1100°C, 1300°C, 1500°C, *α* increased to 102–120, 122–141, and 219–304, respectively. In contrast, the fabrics annealed at 1300°C exhibited a much higher *α* of 381–491. Overall, increasing the UHS temperature strengthened the EMW attenuation capability of the fabrics.

In addition to *α*, impedance matching plays a crucial role in EMA performance and can be evaluated using |*Z_in_*/*Z_0_* |. *Z_in_* was calculated using the following equation [60, 61]:

(6) $$Z_{in}=Z_{0}\;\sqrt{\frac{\mu_{r}}{\varepsilon_{r}}}\tan\;h\left[j\frac{2\pi fd}{c}\sqrt{\mu_{r}\varepsilon_{r}}\right]$$

here, *Z_in_* and *Z_0_* are the input impedance of the material and the free‐space impedance, respectively; *d* is the thickness of the material; and *c* and *f* are the speed of light and frequency in free space, respectively. When |*Z_in_*/*Z_0_* | approaches 1, the material exhibits better impedance matching, allowing more EMW to enter the material. Generally, |*Z_in_*/*Z_0_* | values between 0.8 and 1.2 are considered to satisfy impedance matching requirements. The raw fabrics exhibited poor impedance matching, as shown in Figure S14a. The fabrics after UHS at 1100°C or 1300°C showed a broad frequency range with |*Z_in_*/*Z_0_* | values within 0.8–1.2, indicating good impedance matching (Figure 3f,g). In contrast, for the fabrics after UHS at 1500°C or annealing at 1300°C, |*Z_in_*/*Z_0_* | values mostly fell outside 0.8–1.2, indicating poor impedance matching (Figure 3h and Figure S14b). Overall, the fabrics after UHS at 1100°C or 1300°C exhibited good impedance matching, favoring efficient EMW absorption.

According to transmission line theory, the reflection loss (*RL*) was calculated as follows [62, 63]:

(7) $$RL=20\log\left|\frac{Z_{in}-Z_{0}}{Z_{in}+Z_{0}}\right|$$

*RL*_min_ and EAB are two key parameters for evaluating the EMA performance [51]. An *RL* below −10 dB indicates that >90% of EMW are absorbed; the corresponding frequency range is defined as the EAB [64, 65]. For the fabrics pyrolyzed at 800°C, *RL*_min_ is only −4.30 dB, indicating weak EMW attenuation and substantial EMW transmission (Figure S15a). After UHS at 1100°C, the fabrics achieved an *RL*_min_ of −77.09 dB and an EAB of 9.79 GHz, covering nearly the entire X‐ and Ku‐bands (Figure 3i). After UHS at 1300°C, the fabrics exhibited the best EMA performance, with an *RL*_min_ of −83.33 dB and an EAB of 9.80 GHz, fully covering the X‐ and Ku‐bands (Figure 3j). In contrast, the fabric after UHS at 1500°C or annealing at 1300°C showed weaker EMW absorption performance (Figure 3k and Figure S15b). In short, the SiBCNZr fiber fabrics after UHS at 1300°C exhibited excellent EMA performance. Compared with other ceramic‐based EMA materials (e.g., ceramic fibers, ceramic aerogels, and bulk ceramics), the SiBCNZr fabrics after UHS at 1300°C exhibited better EMA performance in both *RL*_min_ and EAB (Figure 3l and Table S1).

Furthermore, to evaluate the application potential of the fabrics as high‐temperature electromagnetic wave absorbers, the temperature‐dependent electromagnetic parameters and electromagnetic wave absorption performance of the SiBCNZr fabrics after UHS at 1300°C were measured in the X‐band from 100°C to 500°C (Figures S16–S18). Both the real and imaginary parts of the complex permittivity increased with increasing temperature, resulting in an enhanced attenuation constant and improved electromagnetic energy dissipation capability at elevated temperature (Figure S16). Meanwhile, the *|Z_in_/Z_0_|* values indicate that favorable impedance matching can be maintained at elevated temperature by reducing the matching thickness (Figure S17). Consequently, the fabric exhibited excellent electromagnetic absorption performance over the entire tested temperature range (Figure S18). Notably, even at 500°C, the fabric retained an *RL*_min_ of −66.02 dB and an EAB of 4.20 GHz, demonstrating promising high‐temperature electromagnetic wave absorption capability.

**FIGURE 4:**
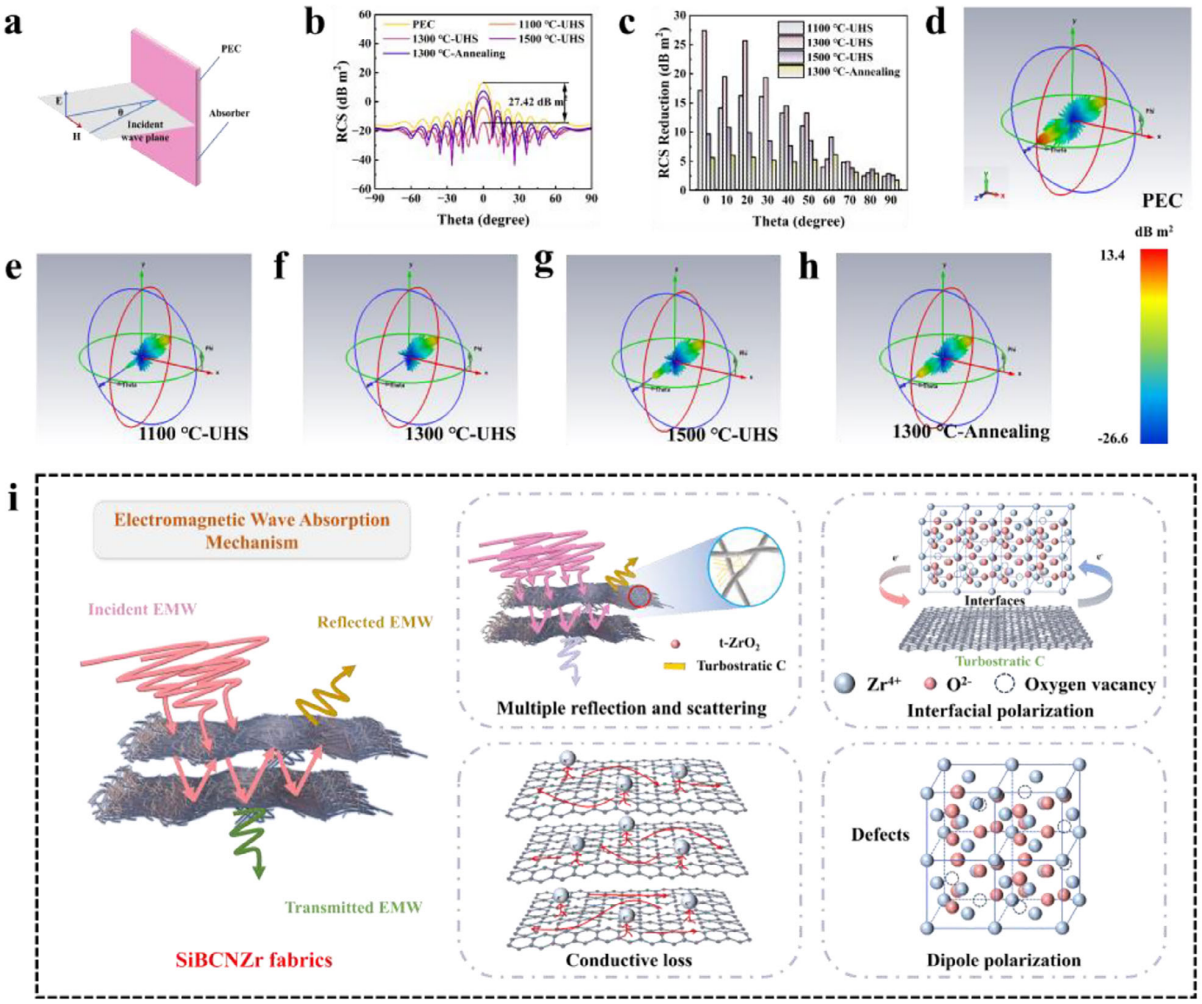
Radar cross‐section (RCS) simulations and EMA mechanism of the SiBCNZr fabrics. (a) RCS simulation model implemented in CST. (b) Simulated RCS versus angle curves at 11.85 GHz for a perfect electric conductor (PEC) plate and the fabrics after UHS or annealing. (c) The reduction in RCS of the fabrics after UHS and annealing. (d–h) 3D RCS patterns at 11.85 GHz for the PEC plate and the fabrics after UHS or annealing. (i) Schematic illustration of the EMA mechanism in the SiBCNZr fabrics.

To further assess the practical performance of the SiBCNZr fabrics, the radar cross section (RCS) was simulated using CST Studio Suite 2023. The model consisted of a perfect electric conductor (PEC) plate with and without a SiBCNZr fabric coating (Figure 4a). Differences among the samples were visualized using 2D RCS distribution patterns (−90° to 90°) and 3D far‐field radiation patterns at 11.85 GHz for a bare PEC plate and PEC plates coated with SiBCNZr fabrics (Figure 4b–h). The bare PEC plate exhibited strong radar scattering (Figure 4d). For the SiBCNZr fabrics treated by UHS, the scattering intensity first decreased and then increased, consistent with the measured *RL* trends (Figure 4e–g). Notably, the fabric after UHS at 1300°C demonstrated the lowest scattering intensity, with a maximum RCS reduction of 27.4 dB m^2^, indicating the best EMW stealth capability (Figure 4b,c,f). In contrast, the fabrics annealed at 1300°C showed a stronger scattering signal, indicating weaker EMW stealth capability than the fabrics after UHS at 1300°C (Figure 4h). In short, the SiBCNZr fabrics after 1300°C UHS exhibited the best EMW stealth performance and show promise for aerospace applications.

Based on the above analysis, the EMA mechanisms of SiBCNZr fabrics after UHS and annealing have been summarized below. The fabric pyrolyzed at 800°C exhibited an amorphous structure (Figure S6) and a low electrical conductivity of 0.091 S·m^−1^, consistent with insulating behavior. When incident EMW impinges on the insulating fabrics, it largely penetrates the fabrics. Because the fabric lacks effective EMW loss components, the incident EMW is not efficiently dissipated, resulting in weak EMW attenuation. Therefore, the raw fabrics behave as EMW‐transparent materials.

After UHS at 1100°C, a small amount of turbostratic C nanoclusters and defect‐rich *t*‐ZrO_2_ nanocrystals (∼4 nm) precipitated within the fabric (Figure 2a–d). When the UHS temperature increased to 1300°C, more turbostratic C nanoclusters and defect‐rich *t*‐ZrO_2_ nanocrystals (∼6 nm) precipitated from the amorphous insulating matrix (Figure 2e–h), which increased carrier and dipole concentrations and thereby enhanced conduction and polarization losses for EMW (Figure 4i). Meanwhile, abundant heterointerfaces formed among the turbostratic C nanoclusters, defect‐rich *t*‐ZrO_2_ nanograins, and the amorphous fibrous matrix, promoting interfacial polarization loss for EMW. Moreover, multiple reflections within the fibrous network further enhanced EMW attenuation. Conduction loss, dipolar polarization loss, interfacial loss, and multiple reflections acted synergistically to EMW attenuation. More importantly, an appropriate amount of turbostratic C nanoclusters and defect‐rich *t*‐ZrO_2_ nanocrystals dispersed in the amorphous insulating matrix improved impedance matching, enabling broadband (EAB = 9.8 GHz) and strong (*RL*_min_ = −83.33 dB) EMA performance (Figure 3j). In conclusion, based on the precise control of microstructure achieved through UHS, it is possible to achieve a significant balance between EMW attenuation and impedance matching, thereby obtaining broadband and strong EMA performance.

For the fabrics after UHS at 1500°C or annealing at 1300°C, excessive *t*‐ZrO_2_ nanograins and turbostratic C nanoclusters formed within the nanofibers, which further increased conduction loss, dipolar polarization loss, and interfacial loss, yielding strong EMW attenuation. However, the excessive fraction of EMW loss components deteriorated impedance matching. As a result, these fabrics exhibited poor EMA performance. In conclusion, the rapid and controllable microstructural evolution enabled by UHS balances EMW attenuation and impedance matching, enabling broadband and strong EMA performance.

**FIGURE 5:**
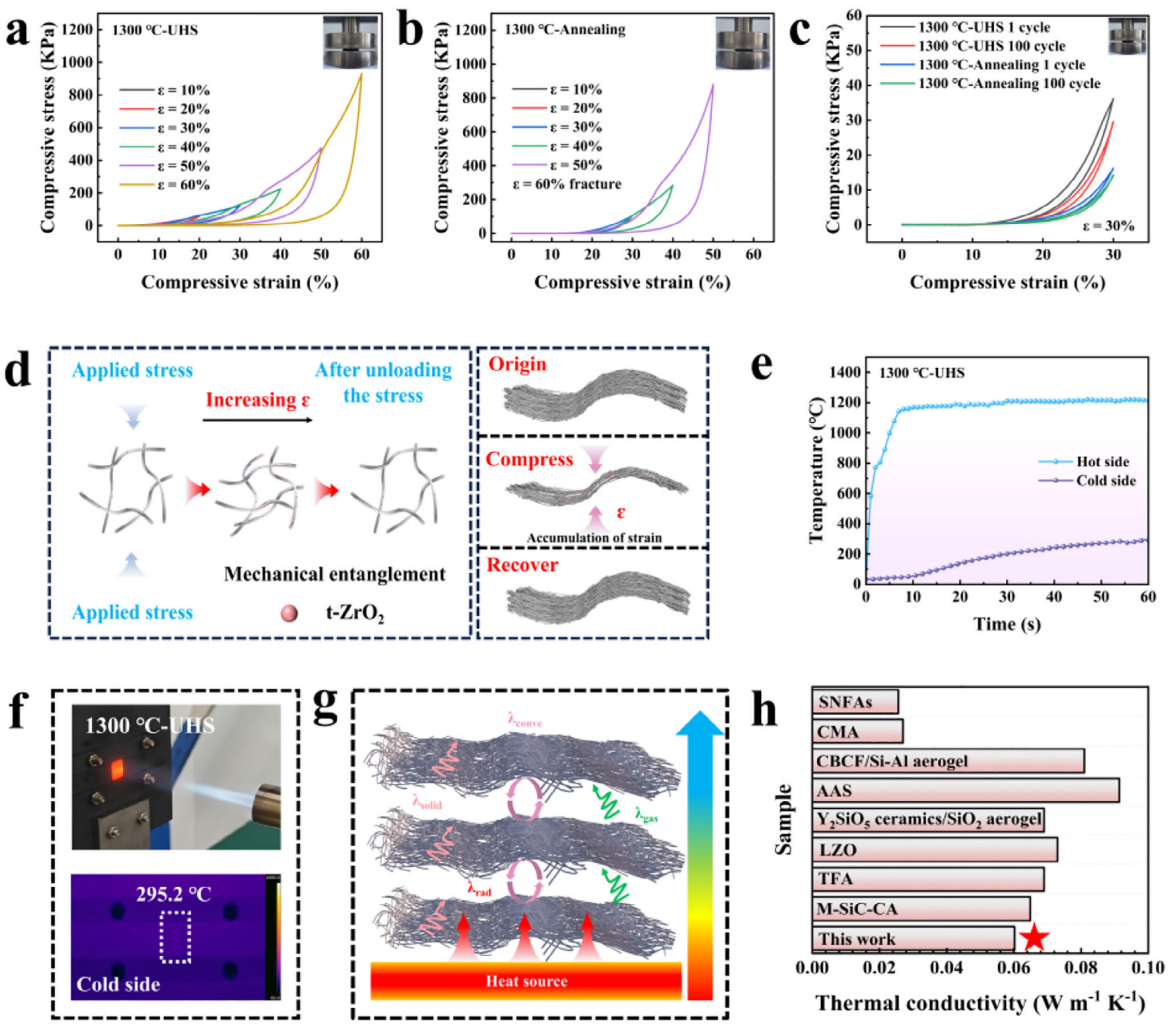
Compression resilience and high‐temperature thermal insulation of the SiBCNZr fabrics. (a, b) Stress–strain curves of the SiBCNZr fabrics after UHS at 1300°C and after annealing at 1300°C. (c) Stress–strain curves recorded at the 1st and 100th cycles under 30% compressive strain for the fabrics after UHS at 1300°C and after annealing at 1300°C. (d) Schematic illustration of the compressive‐resilience mechanism of the fabrics. (e) Temperature‐time curves. (f) Infrared thermal and optical images of the front and back surfaces of the fabrics after UHS at 1300°C during exposure to a butane torch. (g) Schematic illustration of the thermal insulation mechanism. (h) Comparison of thermal conductivity with representative materials reported in the literature.

To quantitatively evaluate elastic behavior, the SiBCNZr fabrics after UHS/annealing at 1300°C were compressed to strains of 10%–60% (Figure 5a,b). The SiBCNZr fabrics after UHS at 1300°C exhibited rapid shape recovery after 60% compressive strain, demonstrating outstanding elastic resilience. The fabrics maintained complete shape recovery across all strain levels and reached a maximum stress of 930 kPa at 60% strain without structural failure (Figure 5a). In contrast, the fabrics after annealing at 1300°C showed prompt recovery below 60% strain but fractured at 60% strain (Figure 5b). The fabric after UHS at 1300°C also demonstrated superior fatigue resistance, maintaining stable performance over 100 compression cycles at 30% strain. After 100 cycles, the maximum stress remained at 29.5 kPa (81.46% of the initial value) (Figure 5c), highlighting remarkable durability under repeated compression. Although the fabric after annealing at 1300°C showed elastic recovery at 30% strain, its maximum stress after 100 cycles was only 14.2 kPa, lower than that of the fabric after UHS at 1300°C. Therefore, the fabrics after UHS at 1300°C demonstrated superior compressive resilience compared with the annealed fabrics. To further quantify elastic recovery, the cyclic recovery ratio (*R*) was calculated as follows [66]:

(8) $$R=\left(1-\frac{\varepsilon_{unrec}}{\varepsilon_{max}}\right)\times 100\%$$

where *ε_max_* and *ε_unrec_* are the maximum strain and unrecoverable strain, respectively. Both fabrics maintained a cyclic recovery ratio of 100% after 100 compression cycles, demonstrating excellent recoverability and fatigue‐resistant elastic behavior.

The mechanical elasticity of SiBCNZr fabrics is governed by the deformability of individual fibers and the weak inter‐fiber friction [67] (Figure 5d), as follows: (1) Defects promote deformation via dislocation slip. Defects in *t*‐ZrO_2_ nanograins and turbostratic C nanoclusters facilitate dislocation slip, thereby increasing the deformability [68]. (2) Heterointerfaces promote fiber bending by enabling interfacial sliding and grain rotation. Abundant heterointerfaces among the *t*‐ZrO_2_ nanograins, turbostratic C nanoclusters, and amorphous fibrous matrix can facilitate grain rotation and interfacial accommodation, enhancing the deformability of individual fibers [69]. (3) Nanoscale fibers exhibit improved bendability. One‐dimensional SiBCNZr fibers with diameters of 100–700 nm can accommodate large bending deformation by axial accumulation of small local strains, therefore improving fiber deformability [70]. (4) Weak inter‐fiber and mechanical entanglement promotes elastic resilience by allowing the fabrics to recover after high compressive stain [71, 72]. In summary, the outstanding elastic resilience of SiBCNZr fabrics arises from the defect‐assisted dislocation activity, interfacial accommodation, nanoscale fiber diameters, and weak inter‐fiber friction/entanglement.

To evaluate thermal insulation under extreme high‐temperature conditions, SiBCNZr fabrics after UHS at 1300°C were exposed to a butane flame, and the front‐ and back‐surface temperatures were recorded using two infrared cameras (Figure 5e,f). When the butane torch was brought close to the fabric, the front‐surface temperature rapidly increased to ∼1200°C within 10 s and then stabilized at ∼1200°C. In contrast, the back‐surface temperature also increased rapidly but remained below 300°C throughout the test. For the fabrics with a thickness of 5 mm, the temperature difference between the front and back surfaces reached ∼900°C, corresponding to a temperature gradient of 180°C mm^−1^, demonstrating excellent high‐temperature thermal insulation.

The thermal insulation performance of the SiBCNZr fabrics was closely linked to their thermal conductivity. The thermal conductivity of the fabric after UHS at 1300°C was as low as 0.0603 W m^−1^ K^−1^, comparable to that of many reported ceramic fiber materials (Figure 5h and Table S2). The low thermal conductivity of SiBCNZr fabrics arose from suppressed heat transport in both the solid and gas phases, with contributions from solid conduction (*λ_solid_*) and gas conduction (*λ_gas_*) (Figure 5g) [73, 74]. (1) Nanoscale grains reduced *λ_solid_*. The *t*‐ZrO_2_ nanograins in the fabrics after UHS at 1300°C had an average grain size of ∼6 nm. Such a small grains size shortened the phonon mean free path (*l_s_*), thereby decreasing *λ_solid_* [75]. (2) Lattice defects reduced the *λ_solid_*. A high density of lattice defects in the *t*‐ZrO_2_ nanograins and turbostratic C nanoclusters (Figure 2f) enhanced phonon scattering and further shortened *l_s_*, leading to a lower *λ_solid_*. (3) Abundant heterointerfaces increased interfacial phonon scattering and reduced *λ_solid_*. Numerous heterointerfaces among the *t*‐ZrO_2_ nanograins, turbostratic C nanoclusters, and the amorphous fibrous matrix promoted interfacial phonon scattering, which also suppressed *l_s_* and *λ_solid_* [76]. (4) The porous architecture introduced *λ_gas_*. Because air has a low thermal conductivity (0.026 W·m^−1^·K^−1^) and the effective thermal conductivity of porous solid strongly depends on the void fraction (porosity), the high porosity of the SiBCNZr fabrics further reduced the total thermal conductivity [77, 78, 79]. In summary, the low thermal conductivity of the SiBCNZr fabrics arose from nanoscale grains, defects in grains, abundant heterointerfaces, and high porosity, which collectively suppressed heat transport in both the solid and gas phases.

## 3 Conclusions

Lightweight, elastic SiBCNZr ceramic nanofiber fabrics with superior EMA and thermal insulation were fabricated via electrostatic spinning, cross‐linking, pyrolysis, and UHS. The precipitation of defect‐rich *t*‐ZrO_2_ nanograins and turbostratic C nanoclusters from an amorphous fibrous matrix was controlled by UHS, enabling a balance between EMW attenuation and impedance matching. As a result, the fabrics after UHS at 1300°C achieved a *RL*_min_ of −83.33 dB and an EAB of 9.8 GHz. Furthermore, the fabrics exhibited elastic resilience up to 60% compressive strain, enabled by defect‐assisted dislocation activity, interfacial accommodation, nanoscale fiber diameters, and weak inter‐fiber friction. Finally, due to nanoscale grains, defects in the grains, abundant heterointerfaces, and high porosity, heat transport in both the solid and gas phases was suppressed, yielding a low thermal conductivity (0.0603 W·m^−1^·K^−1^) and excellent thermal insulation with a temperature difference of ≈900°C. This work provides insights into designing multifunctional materials that integrate EMA with mechanical elasticity and thermal insulation, highlighting their potential for electromagnetic absorption and thermal protection in aerospace applications.

## 4 Experimental Section

### 4.1 Raw Materials

Polyborosilazane (PBSZ), a precursor for SiBCN ceramics, was obtained from the Institute of Chemistry, Chinese Academy of Sciences (ICCAS). Polyacrylonitrile (PAN, M_w_ = 150000) and dicumyl peroxide (DCP) were purchased from Shanghai Aladdin Biochemical Technology Co., Ltd. *N, N‐*Dimethylformamide (DMF, reagent grade, ≥99.8%) and zirconium chloride (ZrCl_4_, Reagent Grade, ≥98%) were purchased from Titan Scientific Co., Ltd. High‐purity argon (Ar, 99.999%) was supplied by Shanghai Haoqi Gas Co., Ltd. All chemicals were used as received without further purification.

### 4.2 Preparation of SiBCNZr Ceramic Nanofiber Fabrics

The SiBCNZr ceramic nanofiber fabrics were prepared by electrostatic spinning, cross‐linking, pyrolysis, and UHS. First, the spinning solution used for electrostatic spinning was prepared by mixing the reagents under stirring. PBSZ (8 g) and DCP (0.08 g) were dissolved in DMF (30 g), and the mixture was stirred for 0.5 h to obtain a homogeneous solution. PAN (1.6 g) was then added, and the solution was stirred for 24 h. ZrCl_4_ (1.44 g) was subsequently added, and the solution was stirred for another 24 h until a yellow precursor solution was obtained. Second, electrostatic spinning was performed using an electrospinning system (JDF 05, Nano Instrument, China). The SiBCNZr fiber preforms were collected by electrostatic spinning under the conditions described below. The applied voltage, feed rate, and needle‐to‐collector distance were set to 23 kV, 1 ml·h^−1^, and 20 cm, respectively. The as‐spun fabrics were cross‐linked at 130°C for 2 h. Then, the cross‐linked fabrics were pyrolyzed at 800°C for 1 h under Ar. Finally, the pyrolyzed fabrics were heat‐treated by UHS using a UHS‐3000 system (Tianjin Zhonghuan, China). UHS was conducted under vacuum at a heating rate of 100°C·s^−1^ to 1100°C, 1300°C, or 1500°C, with a dwell time of 30 s at each temperature. For comparison, the pyrolyzed fabrics were conventionally annealed at 1300°C for 30 s under Ar with a heating rate of 10°C·min^−1^.

### 4.3 Characterization

The phase composition of the SiBCNZr fabrics was analyzed by X‐ray diffraction (XRD; MiniFlex 600‐C, Rigaku, Japan). The graphitization degree of the fabrics was assessed by Raman spectroscopy (inVia‐Reflex, Renishaw, UK). The morphology of the fabrics was observed by the scanning electron microscope (SEM; MAIA3, Tescan, Czech Republic). The microstructure and elemental composition of fabrics were characterized by transmission electron microscopy (TEM; Talos F200S, FEI, USA) equipped with high‐resolution TEM (HRTEM), and selected‐area electron diffraction (SAED). Functional groups were identified by Fourier‐transform infrared spectroscopy (FTIR; Nicolet iS50, Thermo Fisher, China). Electrical conductivity was measured using a Seebeck coefficient/electrical resistance measurement system (ZEM‐3 M10, Advance Riko, Japan). This instrument operates based on the four‐probe method principle, and the conductivity was measured along the in‐plane direction of the fabric. The fabrics was cut into rectangular specimens with dimensions of 15 × 4 mm^2^. For each sample, measurements were performed at least three different positions, and the average value was reported as the final electrical conductivity. The EMA performance in the X‐band (8.2–12.4 GHz) and Ku‐band (12.4‐18 GHz) was evaluated using a vector network analyzer (VNA; N5234, Keysight, USA). The fabrics were cut into specimens of 22.89 × 10.16 mm^2^ for X‐band measurements and 15.82 × 7.9 mm^2^ for the Ku‐band measurements, as shown in Figure S19. The compressive elastic resilience of the fabrics was evaluated via a universal testing machine (AGS‐X, Shimadzu, Japan). Thermal conductivity was determined using a thermal conductivity meter (TC3200, Xiatech, China) based on the transient hot‐wire method. Thermal insulation of the fabrics was evaluated by the temperature difference between the front and back surfaces during exposure to a butane torch. The front‐surface temperature was recorded using an infrared thermal camera (DT‐9897H, CEM, China), and the back‐surface temperature was recorded using another infrared thermal camera (346L, FOTRIC, China).

The average crystallite size of *t*‐ZrO_2_ in the fabrics after UHS and post‐annealing was estimated from the characteristic (101) diffraction peak using the Scherrer equation [80]:

(9) $$D=\frac{K\lambda }{\beta \cos\theta }$$

where *D* is the average crystallite size, *K* is the shape factor, *λ* is the wavelength of Cu *K_α_* radiation, and *β* is the full width at half maximum of the diffraction peak in radians. *θ* is the Bragg diffraction angle. In this work, *K* = 0.89 and *λ* = 0.15406 nm were used.

## Author Contributions

**Fei Yan**: investigation, validation. **Chao Zhao**: formal analysis, methodology, software, investigation. **Qi Ding**: conceptualization, writing – review and editing, funding acquisition, supervision, validation, visualization, investigation. **Jiahao Yang**: investigation, methodology, software, data curation, writing – original draft, formal analysis, visualization. **Wan Jiang**: project administration, resources, supervision, conceptualization. **Zhi Cheng**: investigation, methodology. **Juanjuan Xu**: investigation, validation, data curation. **Yuena Zhang**: methodology, software, investigation. **Rui Wang**: methodology, investigation. **Yuchi Fan**: writing – review and editing, project administration, resources, supervision, funding acquisition, conceptualization.

## Funding

National Natural Science Foundation of China (No. 52302061 and 52572066); Innovation Program of Shanghai Municipal Education Commission (2023ZKZD43).

## Conflicts of Interest

The authors declare no conflicts of interest.

## Supporting information




**Supporting File**: advs76453‐sup‐0001‐SuppMat.docx.

## Data Availability

The data that support the findings of this study are available from the corresponding author upon reasonable request.
