# Supplementary material for: Lightweight, Elastic Ceramic Fabrics for Broadband Electromagnetic Absorption and High Temperature Thermal Insulation
Source: Adv Sci (Weinh). 2026 Jul 13:e76453. Online ahead of print. doi: 10.1002/advs.76453 (PMC13360990; doi:10.1002/advs.76453)
Supplement: Supplementary file 1 — Supporting File: advs76453‐sup‐0001‐SuppMat.docx. [file ADVS-9999-e76453-s001.docx]

**Lightweight, Elastic Ceramic Fabrics for Broadband Electromagnetic Absorption and High-temperature thermal Insulation**

*Jiahao Yang, Qi Ding^*^,* *Juanjuan Xu, Fei Yan, Rui Wang, Yuena Zhang, Chao Zhao, Zhi Cheng, Yuchi Fan^*^, Wan Jiang*

J. Yang, Q. Ding, J. Xu, F. Yan, R. Wang, Y. Zhang, C. Zhao, Z. Cheng, Y. Fan, W. Jiang

State Key Laboratory of Advanced Fiber Materials

College of Materials Science and Engineering

Donghua University

Shanghai 201620, China

E-mail: [dingqi@dhu.edu.cn](mailto:dingqi@dhu.edu.cn); [yuchifan@dhu.edu.cn](mailto:yuchifan@dhu.edu.cn)

**Supplementary equations**

**Cole-Cole semicircle equation**

Based on the Debye theory, the Cole-Cole formula was employed to analyze the polarization relaxation modes^[1]^:

${[\varepsilon^{'}-\frac{\varepsilon_{s}+\varepsilon_{\infty}}{2}]}^{2}+({\varepsilon^{''})}^{2}={[\frac{\varepsilon_{s}-\varepsilon_{\infty}}{2}]}^{2}$ **(S1)**

**Radar cross-section (RCS) simulation using CST**

CST simulations were performed using CST Studio Suite 2023 to calculate the RCS at 11.85 GHz under far-field conditions, thereby assessing the practical electromagnetic wave absorption (EMA) performance of the SiBCNZr fabrics. The model was placed in the x-y plane. A linearly polarized plane wave was incident along the z-axis, with its electric field aligned along the x-axis. A test structure with a fixed square area of 180 × 180 mm^2^ was used. The structure consists of a 5.8 mm-thick composite absorbing layer on a 2 mm-thick Perfect Electric Conductor (PEC) substrate. The scattering direction of the RCS was defined by the angles *θ* and *φ* in spherical coordinates. The RCS value (*σ*) was calculated as follows^[2]^:

$\sigma\left( dB m^{2} \right)=10\log[\frac{4\pi S}{\lambda^{2}}{|\frac{E_{s}}{E_{i}}|}^{2}]$ **(S2)**

where, *S* is the substrate area, *λ* is the incident EMW wavelength, and *E_s_* and *E_i_* are the electric field intensities of the scattered and incident EMWs.

**Supplementary figures**


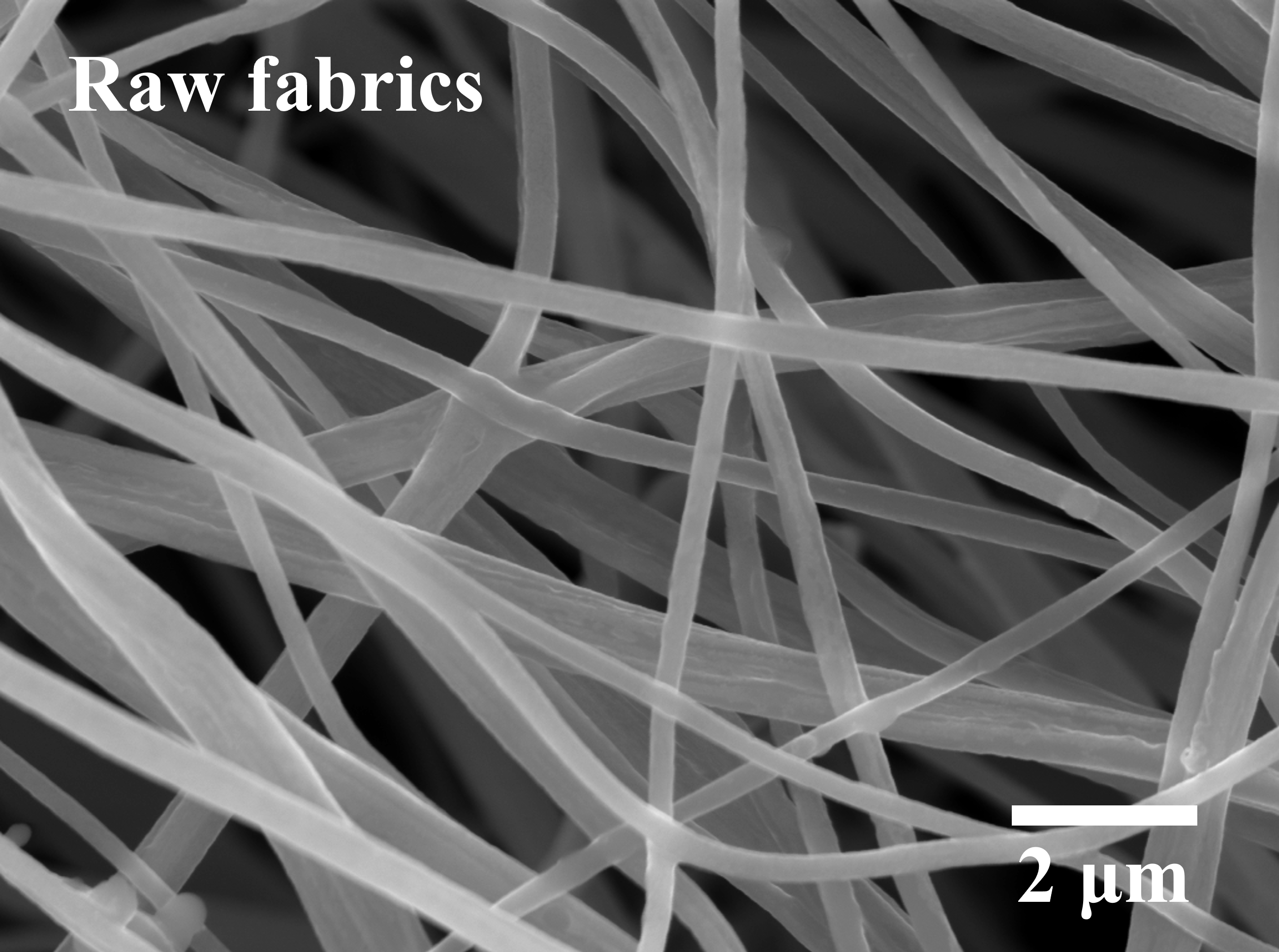
**Figure S1.** a) SEM image of the raw SiBCNZr fabrics.


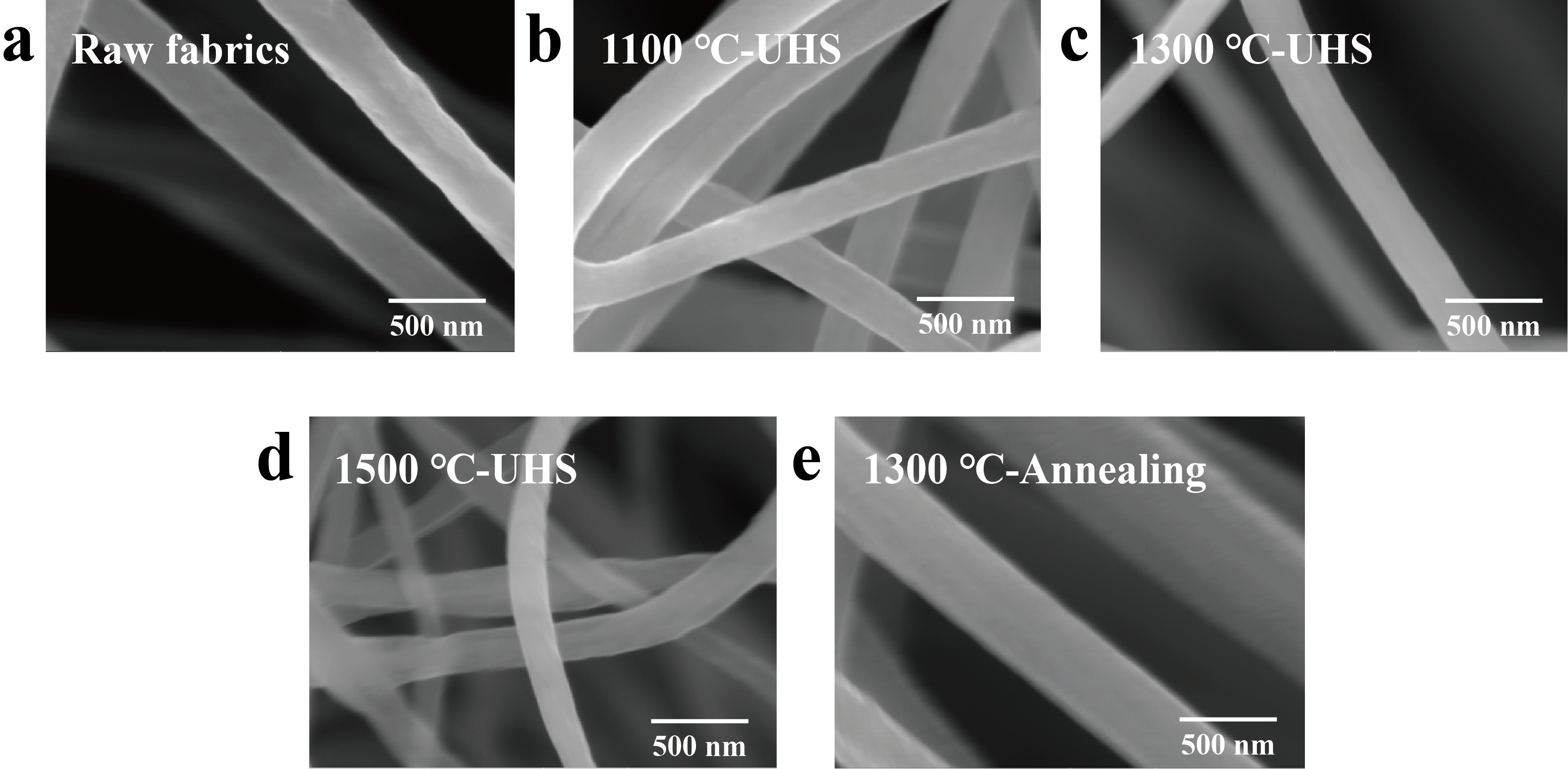
**Figure S2.** High-magnification SEM images of a) raw SiBCNZr fabrics, b-d) SiBCNZr fabrics after UHS at 1100, 1300 and 1500 °C, and e) SiBCNZr fabrics after annealing at 1300 °C.


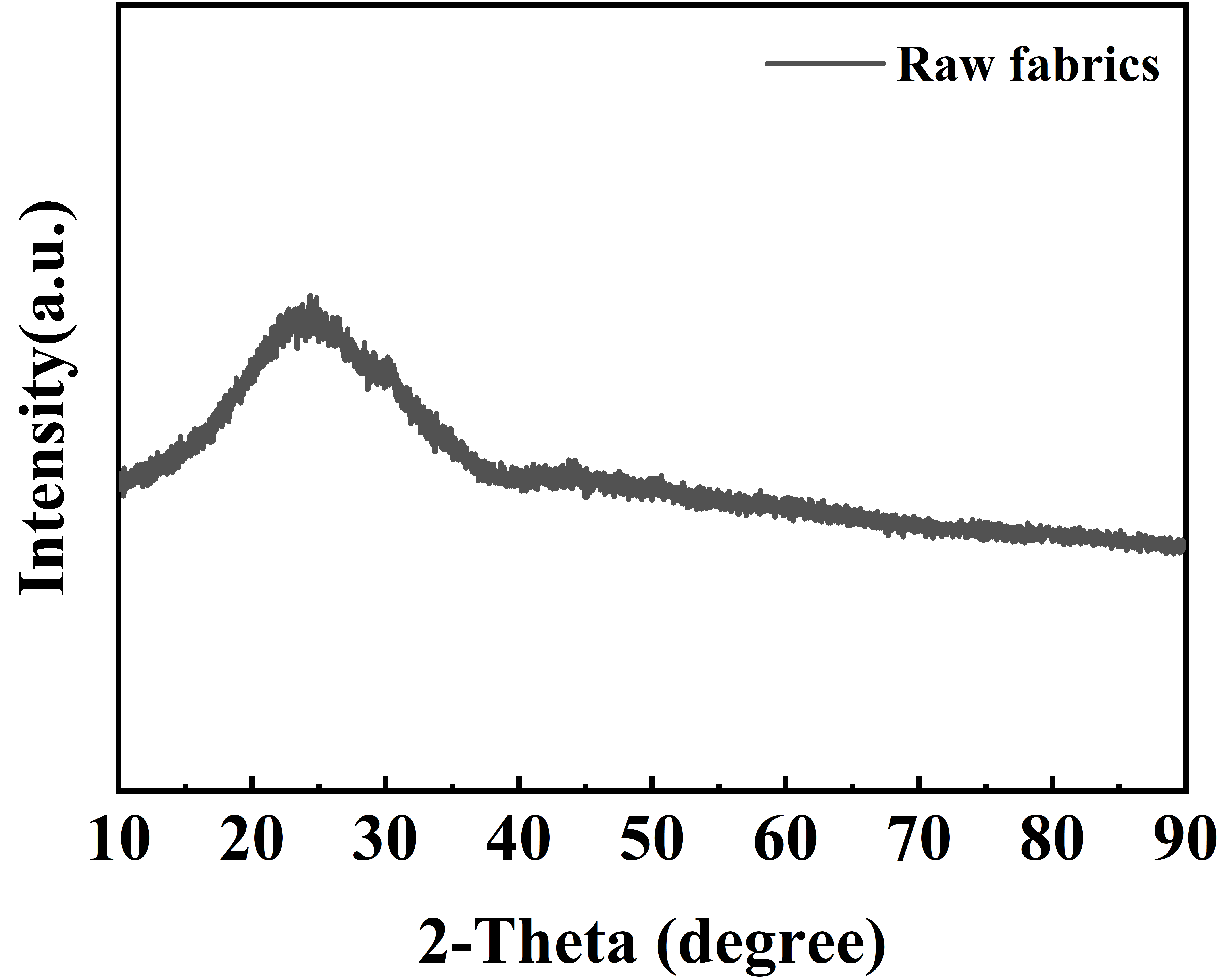
**Figure S3.** XRD patterns of the raw fabrics.


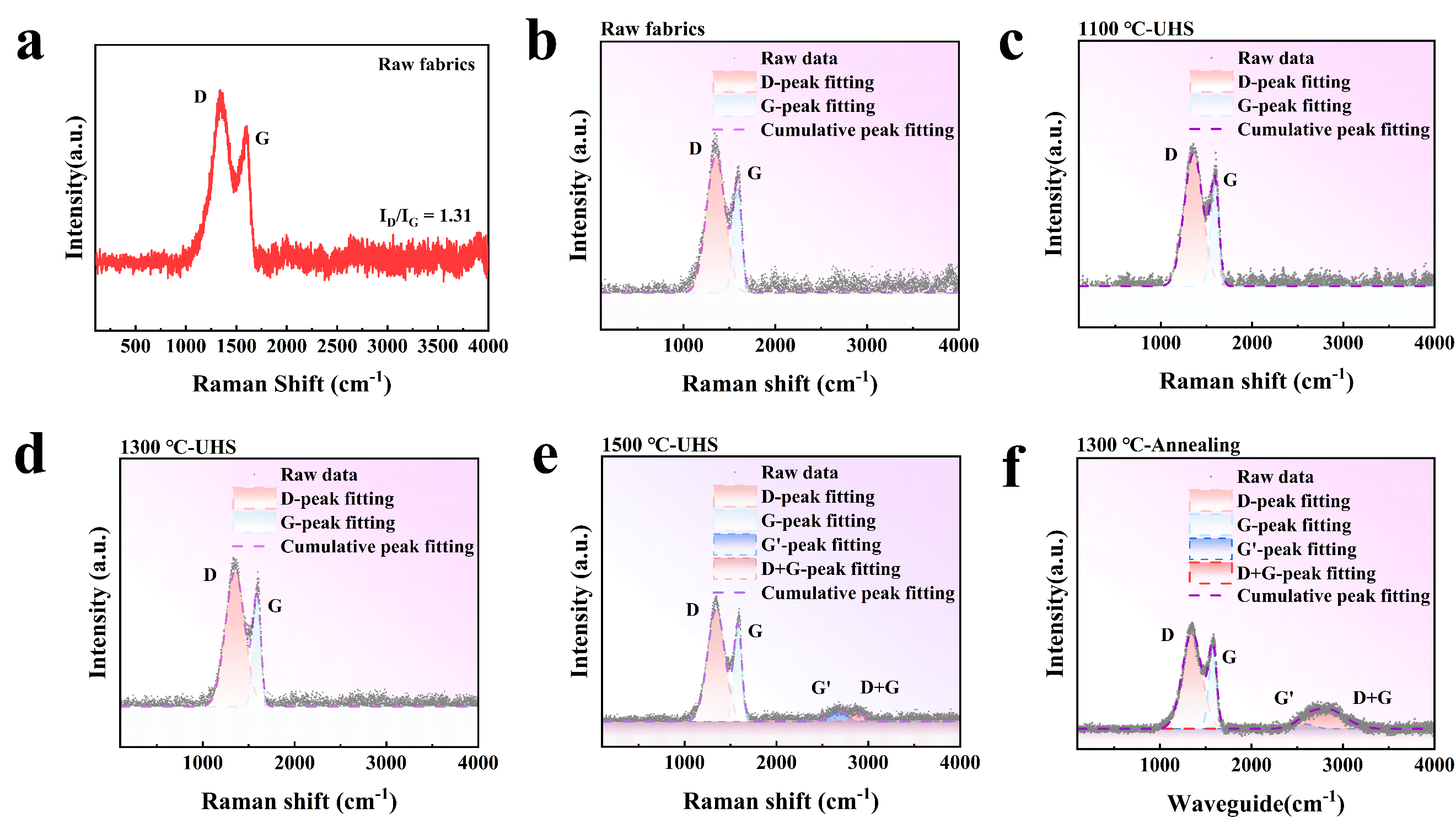
**Figure S4.** a) Raman spectrum of the raw SiBCNZr fabrics. b-f) Fitted Raman spectra of raw SiBCNZr fabrics and fabrics after UHS at 1100, 1300 and 1500 °C, and the fabrics after annealing at 1300 °C.


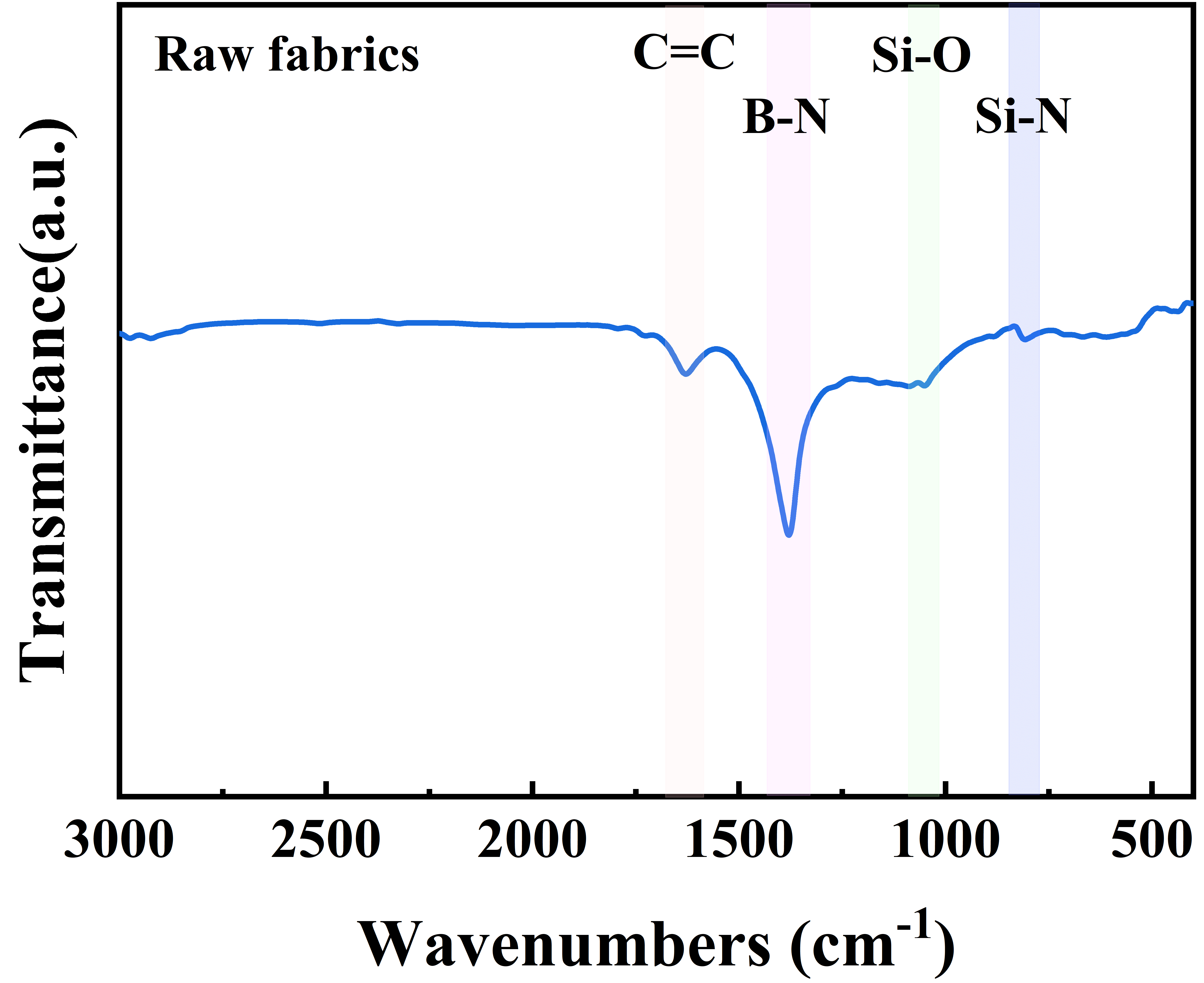
**Figure S5.** FTIR spectrum of the raw SiBCNZr fabrics.


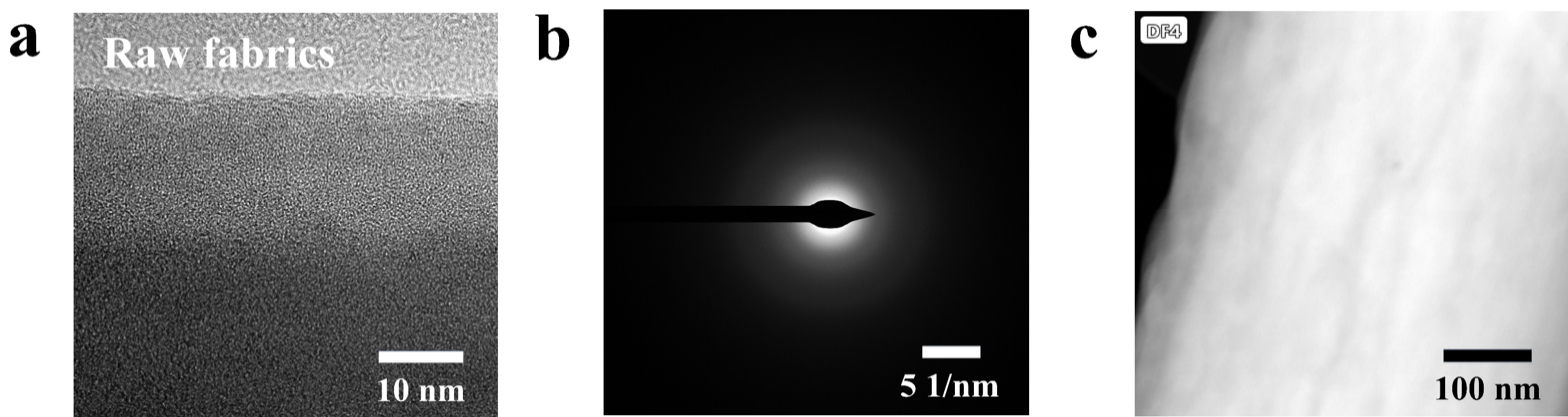
**Figure S6.** a) HRTEM image, b) SAED pattern, and c) STEM image of the raw SiBCNZr fabrics.


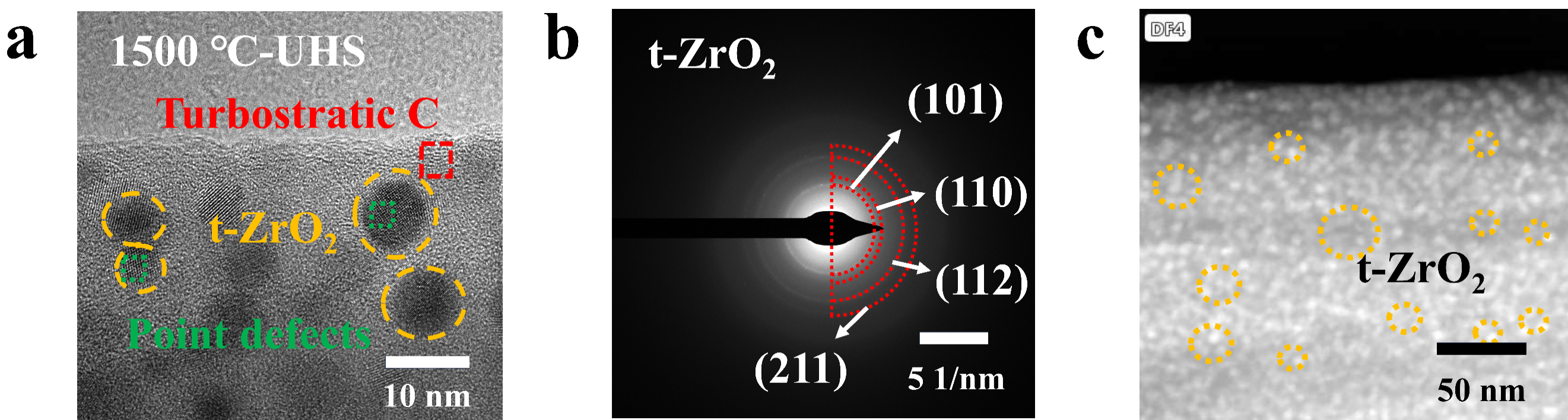
**Figure S7.** a) HRTEM image, b) SAED pattern, and c) STEM image of the SiBCNZr fabrics after UHS at 1500°C.


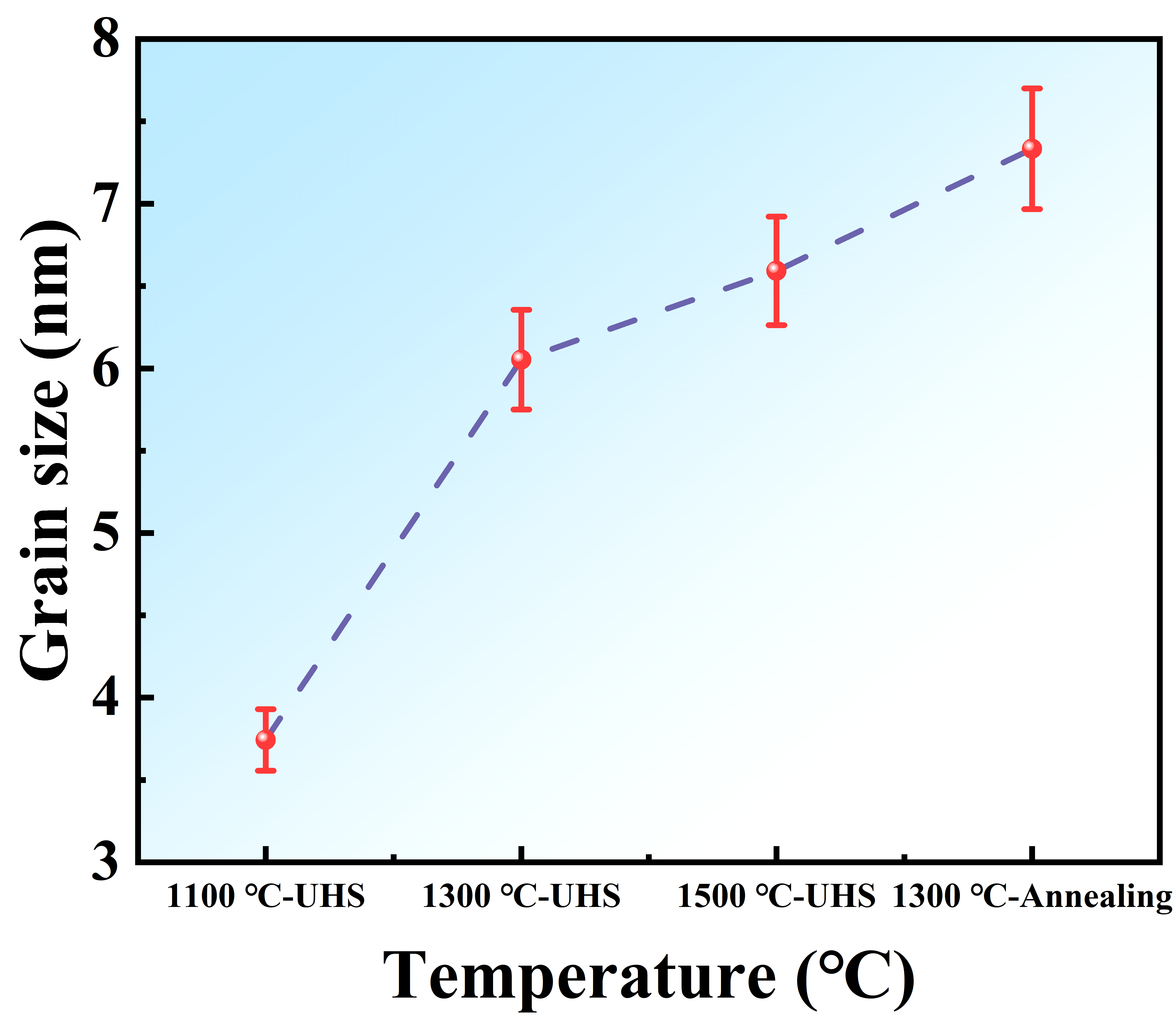
**Figure S8.** The grain sizes of SiBCNZr fabrics after UHS/annealing.


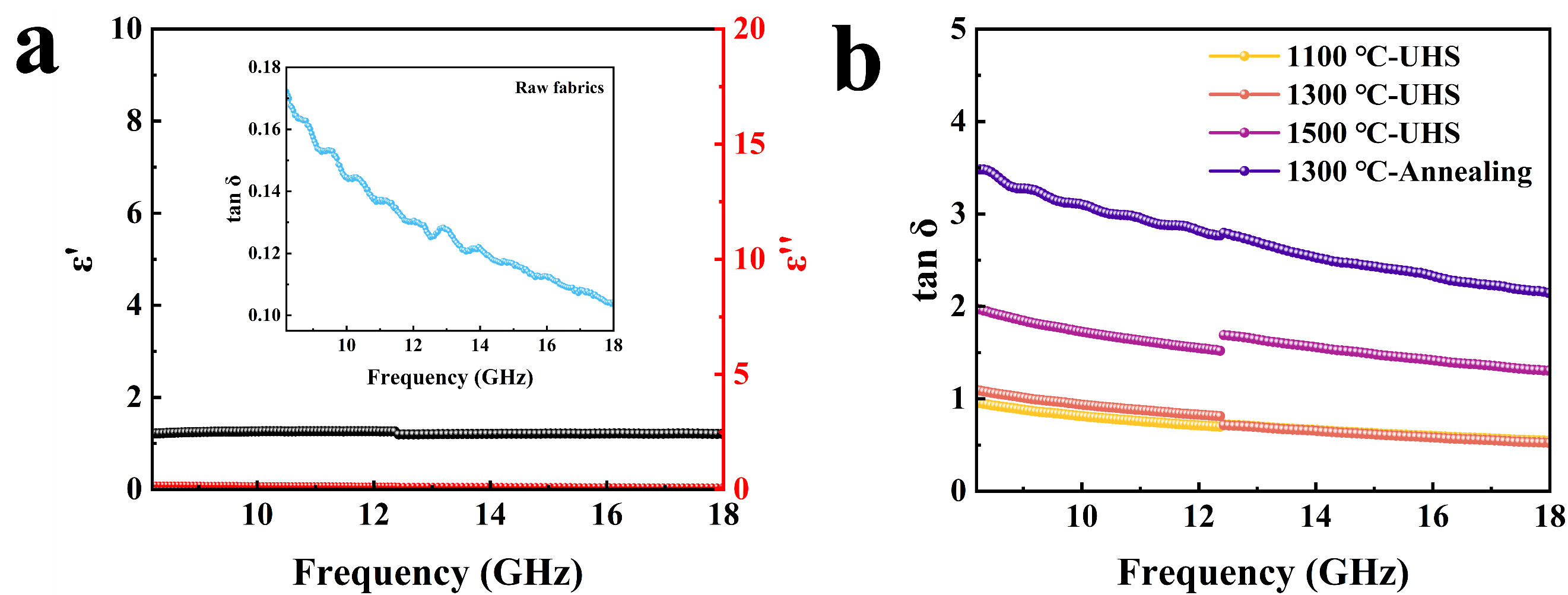
**Figure S9.** a) Relative complex permittivity and Dielectric loss tangent (tan *δ*) of the raw fabrics. b) tan *δ* of the SiBCNZr fabrics after UHS/annealing.


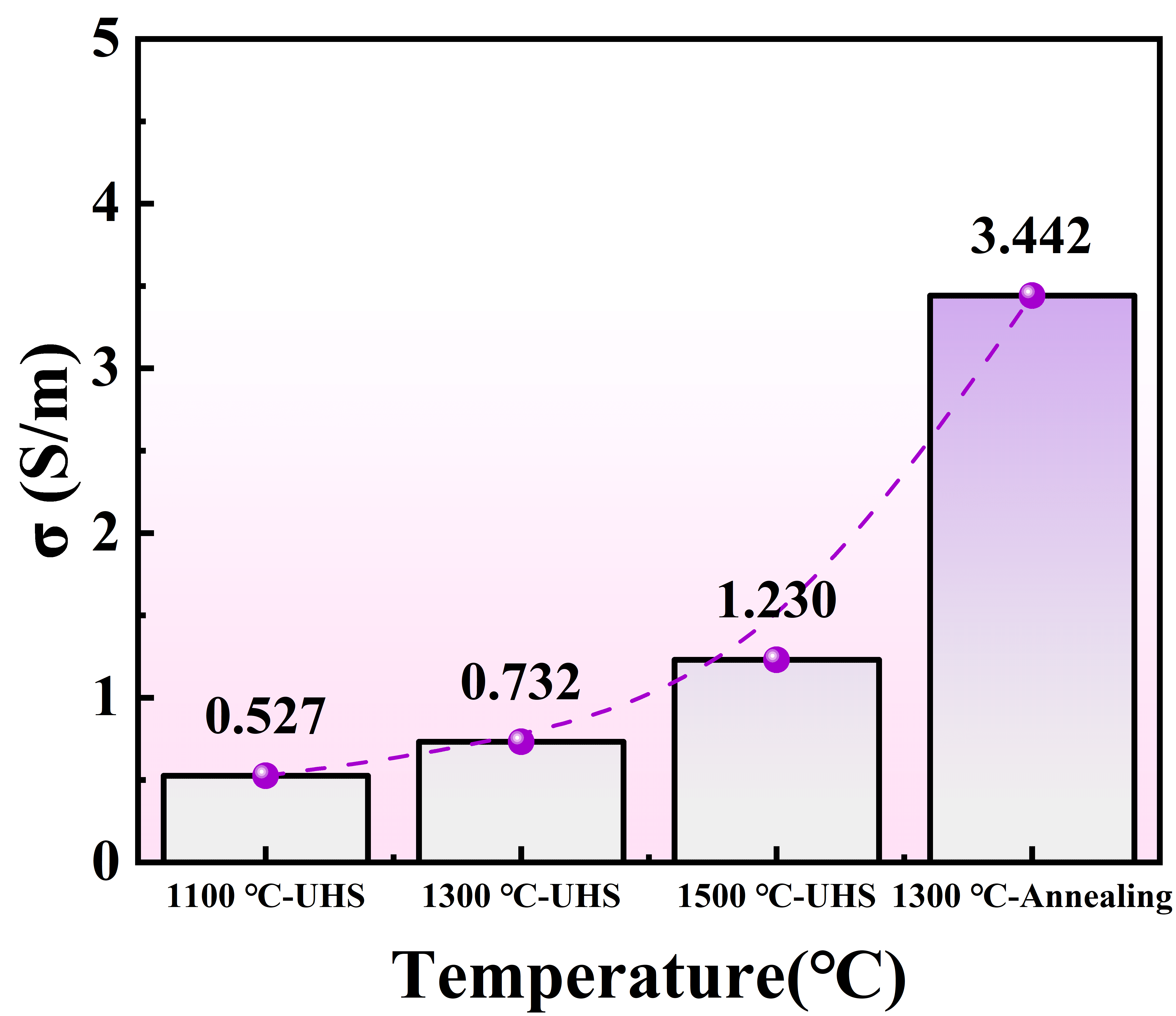
**Figure S10.** Conductivity of the SiBCNZr fabrics after UHS/annealing.


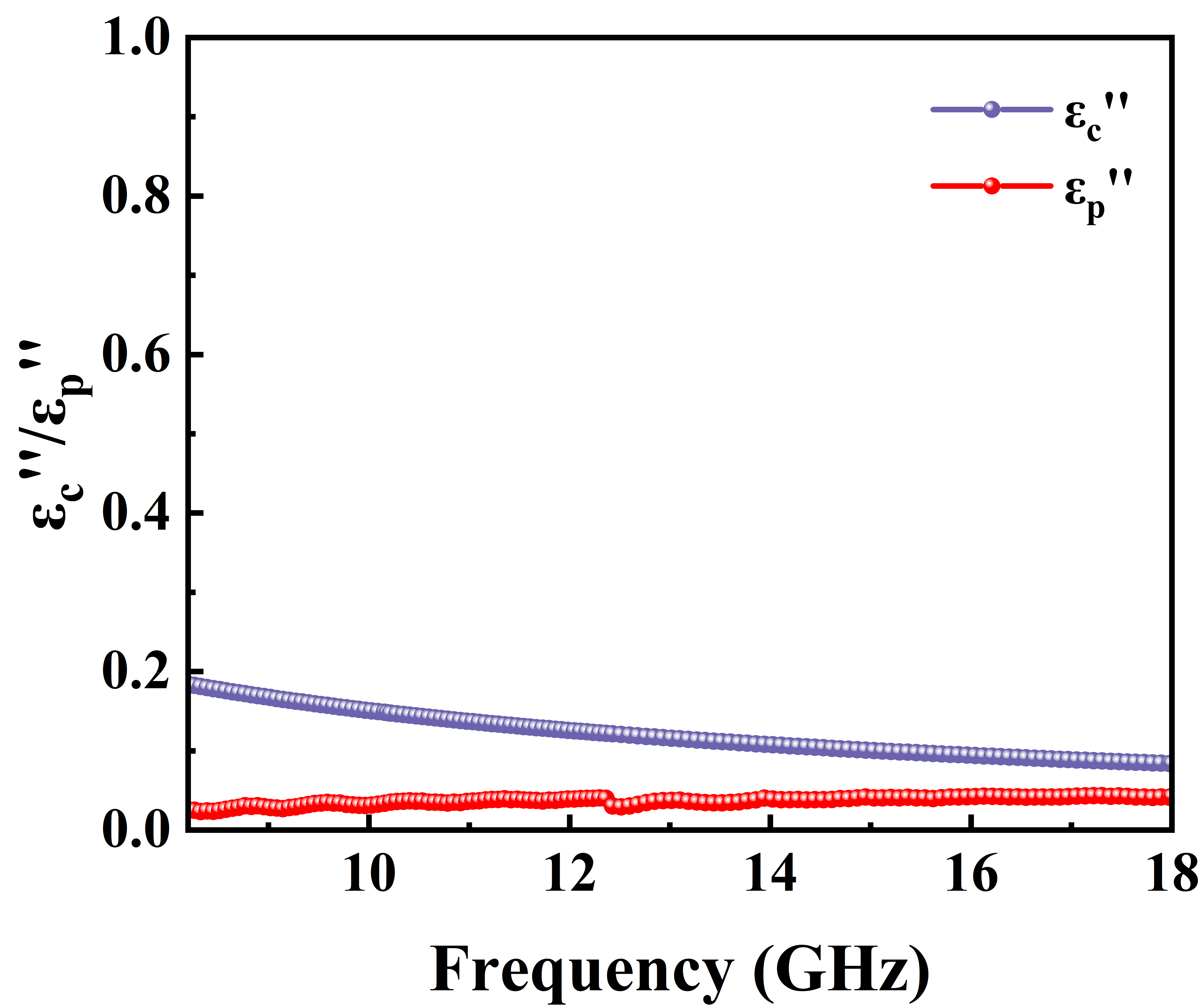
**Figure S11.** Conduction loss (*ε_c_^ʹʹ^*) and polarization loss (*ε_p_^ʹʹ^*) of the raw SiBCNZr fabrics.

**

Figure S12.** Cole-Cole semicircle plots for the SiBCNZr fabrics before and after UHS/annealing: a-e) in the X-band and f-j) in the Ku-band.


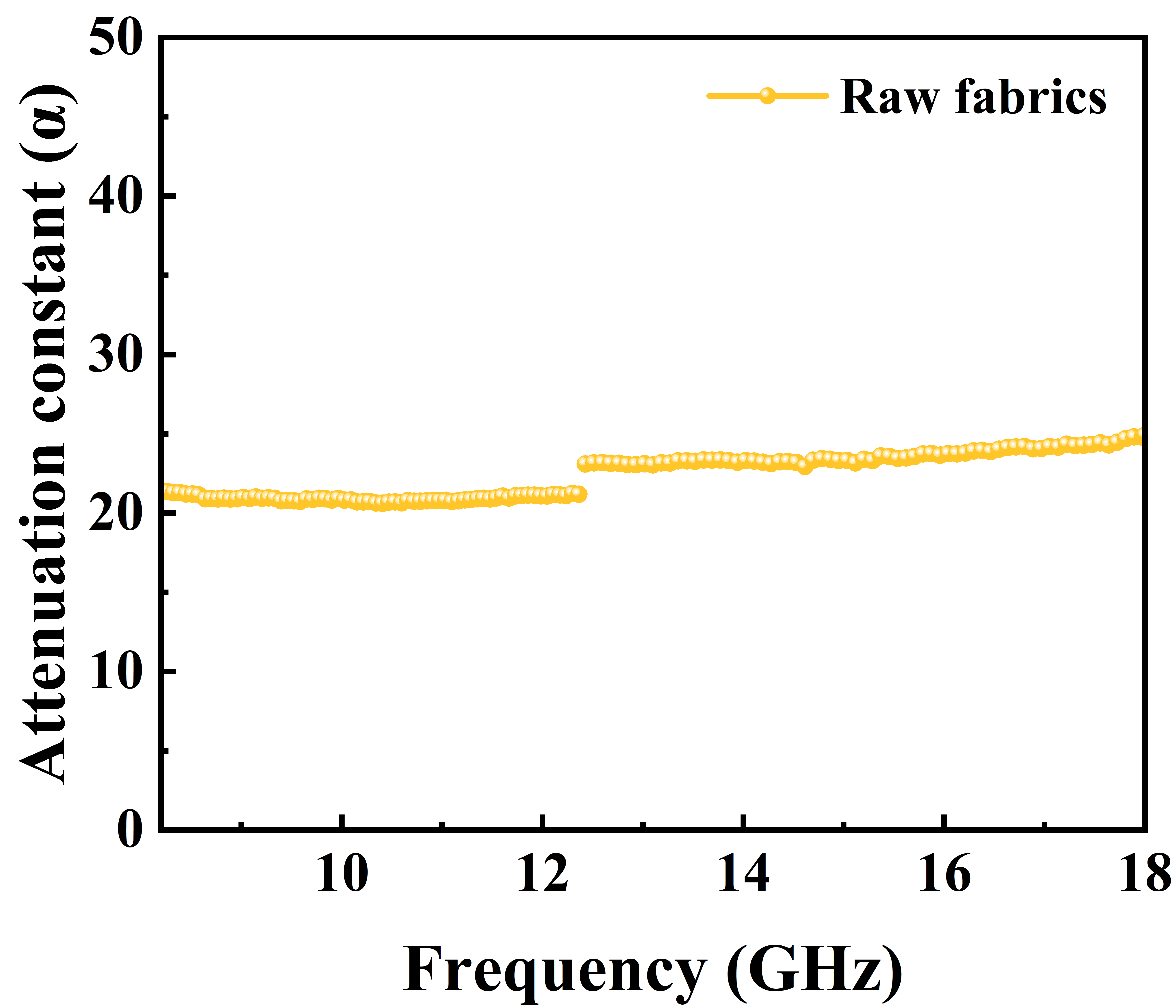
**Figure S13.** Attenuation constant (*α*) of the raw SiBCNZr fabrics.


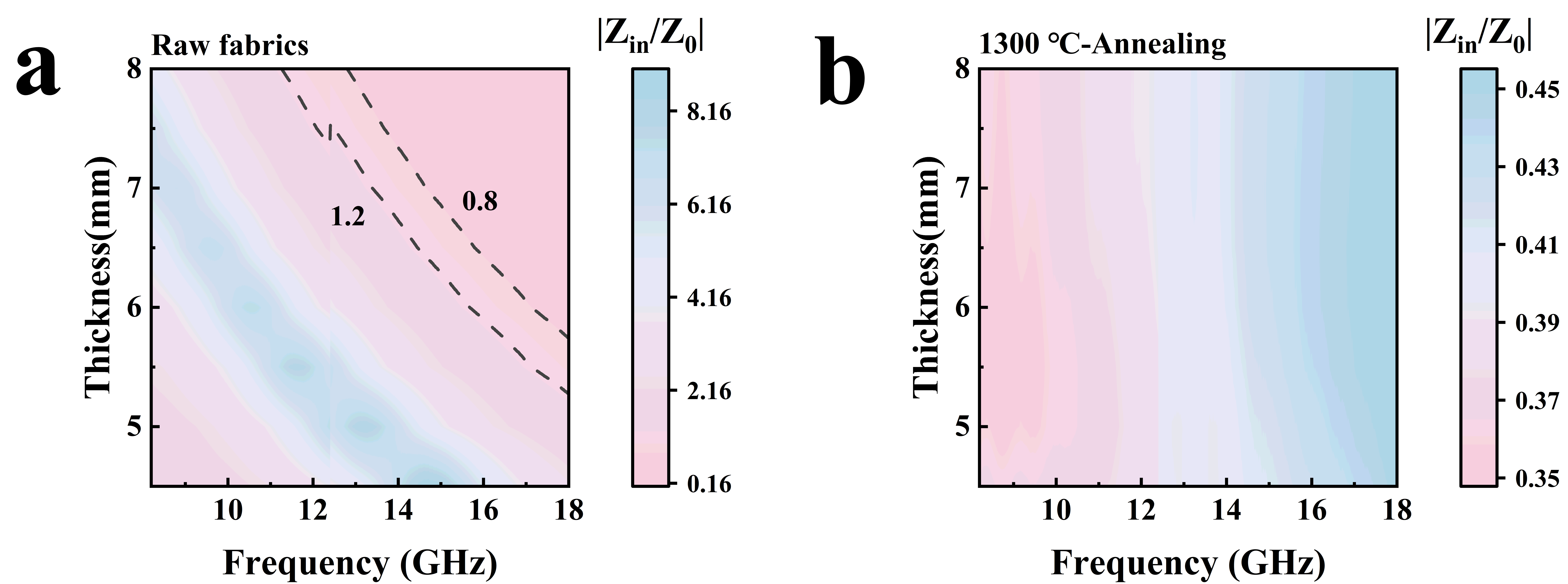
**Figure S14.** |*Z_in_*/*Z_0_*| values of a) the raw SiBCNZr fabrics, and b) the SiBCNZr fabrics after annealing at 1300 °C.


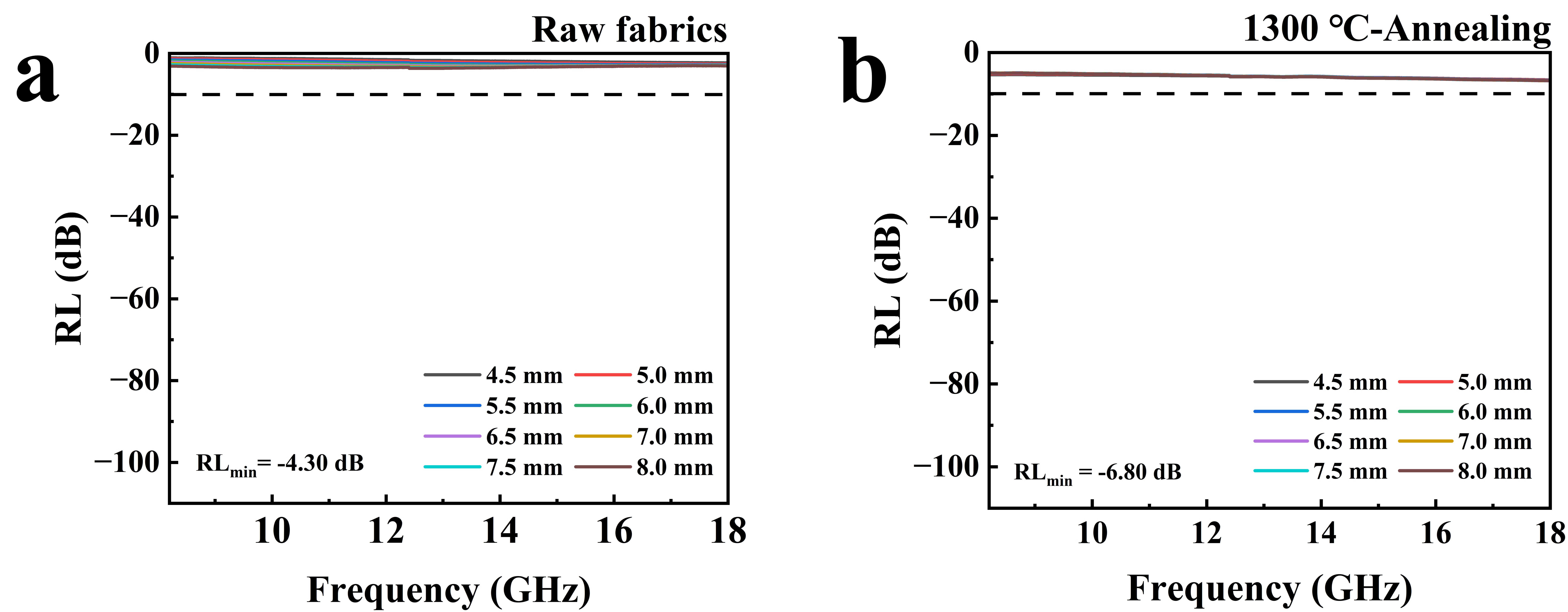
**Figure S15.** *RL* values at different thicknesses of a) the raw SiBCNZr fabrics and b) the SiBCNZr fabrics after annealing at 1300 °C.


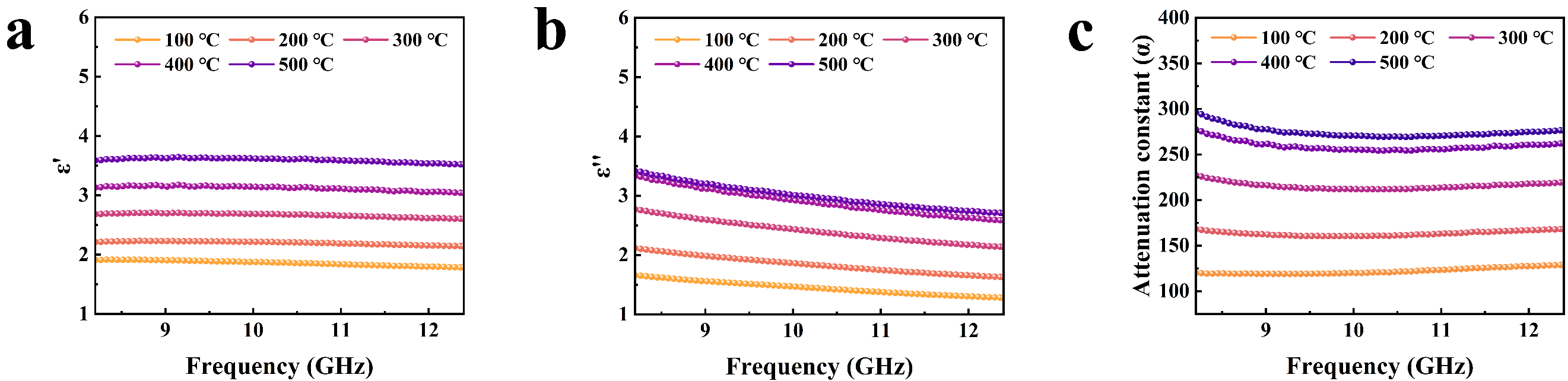
**Figure S16.** Temperature-dependent (a) real permittivity (*εʹ*), (b) imaginary permittivity (*εʹʹ*) and (c) attenuation constant of the SiBCNZr fabrics after UHS at 1300 °C, measured in the X-band at elevated temperatures.


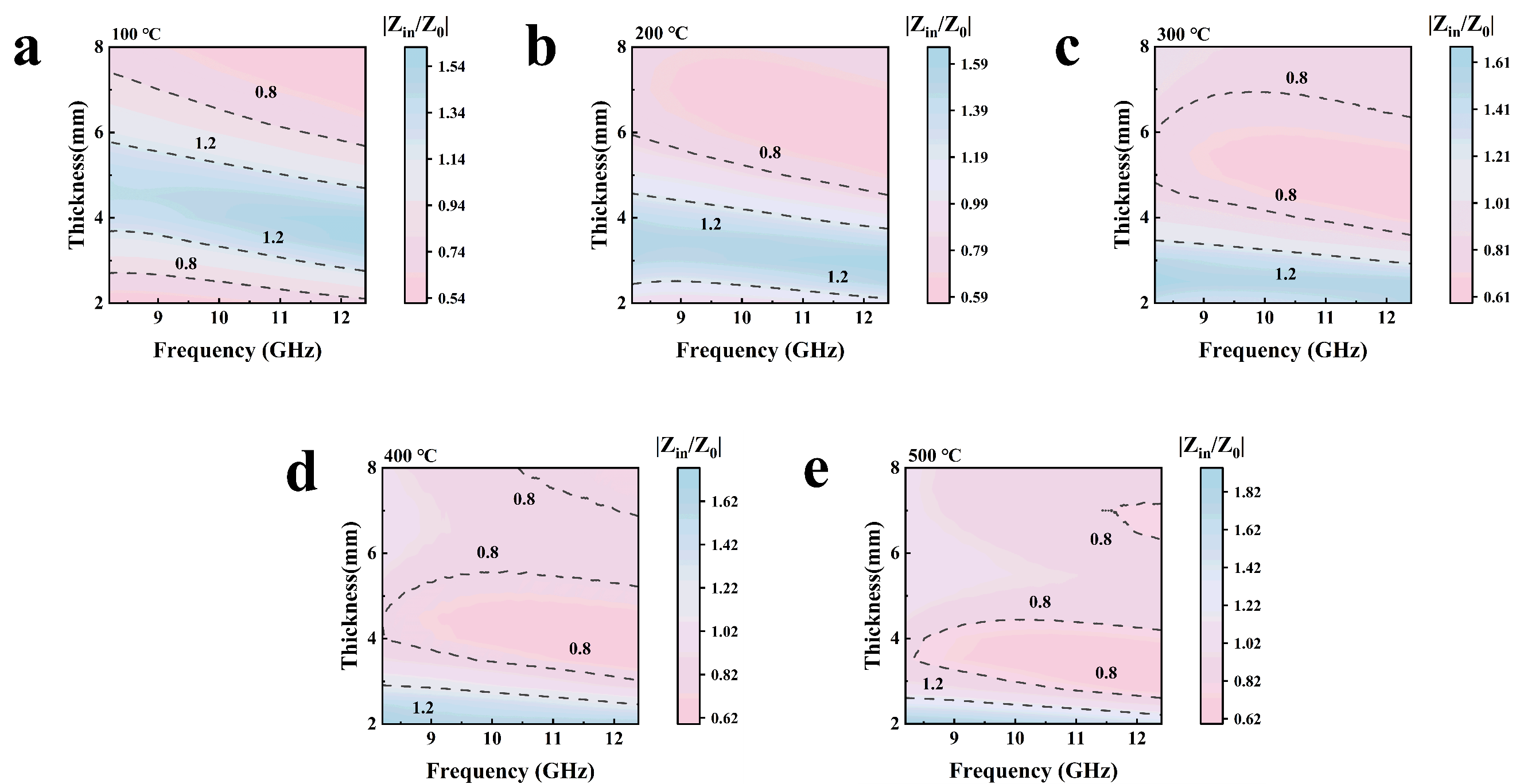
**Figure S17.** Temperature-dependent *|Z_in_/Z_0_|* values of the fabrics after UHS at 1300 °C, measured in the X-band at elevated temperatures.


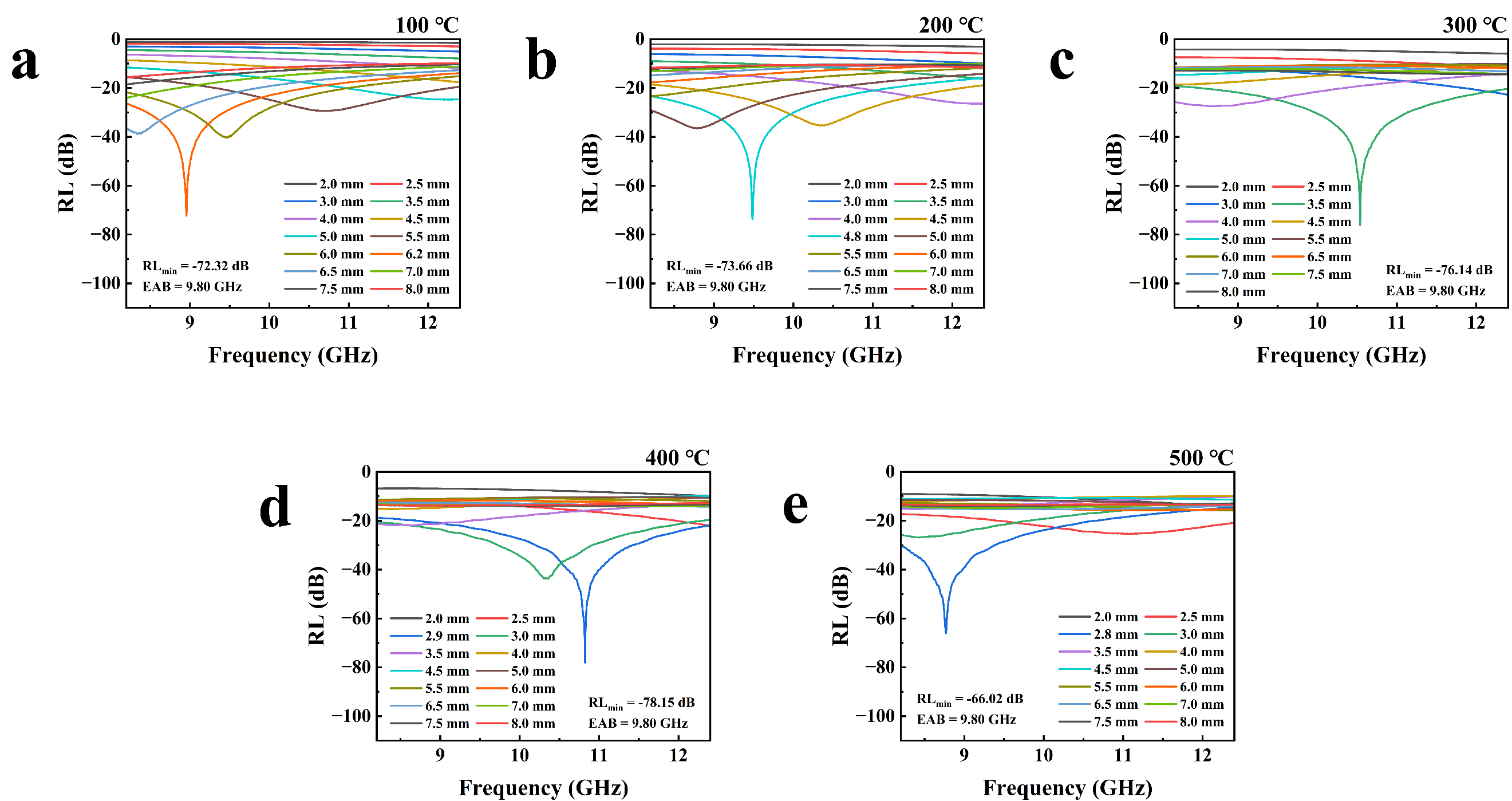
**Figure S18.** Temperature-dependent *RL* values of the SiBCNZr fabrics after UHS at 1300 °C, measured in the X-band at elevated temperatures.

**Figure S19.** Optical photograph of the SiBCNZr fabrics after UHS at 1300 °C prepared for X-band and Ku-band measurements.

**Supplementary tables**

**Table S1.** EMA performance of the ceramic-based materials in this work and in the literature.

| Sample | *RL*_min_ (dB) | EAB (GHz) | Reference |
| --- | --- | --- | --- |
| SiC_f_/FeNi-SiO_2_ | -55.2 | 6.8 | ^[3]^ |
| SiO_2_/rGO/Co-10 | -61.5 | 7.5 | ^[4]^ |
| SiO_2_/rGO/Co-40 | -69.2 | 7.1 |  |
| ZS | -27.1 | 5.5 | ^[5]^ |
| SiCf/Si_3_N_4_-SiC | -51.6 | 8.8 | ^[6]^ |
| SiBNC_x_ | -64.2 | 4.1 | ^[7]^ |
| SiBCN fibers | -54.9 | 4.7 | ^[8]^ |
| Si_x_-O_y_-C_z_/m | -70.4 | 4.3 | ^[9]^ |
| SiBCNZr nanofiber | -83.3 | 9.8 | This work |

**Table S2.** Thermal conductivity of ceramic-based materials in this work and in the literature.

| Sample | Thermal conductivity (W^-1^ m^-1^ K^-1^) | Reference |
| --- | --- | --- |
| M-SiC-CA | 0.065 | ^[10]^ |
| TiO_2_ opacifier/fiber/alumina-based aerogel | 0.069 | ^[11]^ |
| High entropy (LaCeSmEuNd)_2_Zr_2_O_7_ ceramic aerogel | 0.073 | ^[12]^ |
| Y_2_SiO_5_ ceramics/SiO_2_ aerogel | 0.069 | ^[13]^ |
| Unidirectional alumina aerogel | 0.091 | ^[14]^ |
| CBCF/Si-Al aerogel | 0.081 | ^[15]^ |
| Ceramic meta-aerogel | 0.027 | ^[16]^ |
| Silica nanofibrous aerogels | 0.026 | ^[17]^ |
| SiBCNZr fabrics | 0.060 | This work |

**References:**

1. Z. Tang, L. Xu, C. Xie, et al., “Synthesis of CuCo_2_S_4_@Expanded Graphite with Crystal/Amorphous Heterointerface and Defects for Electromagnetic Wave Absorption,” *Nature Communications* 14 (2023): 5951, https://doi.org/10.1038/s41467-023-41697-6.

2. J. Luo, Z. Lv, L. Zhang, Y. Zhong, H. Xu, Z. Mao, “Modulation of Dielectric Behavior in Ceramic-Based Materials for Integrated Electromagnetic Waves Absorption and Thermal Conduction,” *Advanced Functional Materials* 35 (2025): 2420086, https://doi.org/10.1002/adfm.202420086.

3. B. Huang, H. Hu, S. Lim, et al., “Gradient FeNi-SiO_2_ Films on SiC Fiber for Enhanced Microwave Absorption Performance,” *Journal of Alloys and Compounds* 897 (2022): 163204, https://doi.org/10.1016/j.jallcom.2021.163204.

4. B. Li, H. Tian, L. Li, et al., “Graphene-Assisted Assembly of Electrically and Magnetically Conductive Ceramic Nanofibrous Aerogels Enable Multifunctionality,” *Advanced Functional Materials* 34 (2024): 2314653, https://doi.org/10.1002/adfm.202314653.

5. C. Wu, J. Jiang, C. Dong, et al., “Remarkably Enhance the Stealth/Resistance/Mechanical Properties of Silica-Zirconia Ceramic Aerogel by Phase Transitions and Interface Evolution,” *Advanced Functional Materials* 35 (2025): 2505742, https://doi.org/10.1002/adfm.202505742.

6. Z. Xing, X. You, H. Ouyang, et al., “Porous and Lightweight Continuous SiC Fiber Reinforced Si_3_N_4_-SiC Composites for Wide Frequency Electromagnetic Wave Absorption,” *Composites Part B: Engineering* 300 (2025): 112497, https://doi.org/10.1016/j.compositesb.2025.112497.

7. Y. Song, P. Liu, R. Zhou, R. Zhu, J. Kong, “SiBNCx Ceramics Derived from Single Source Polymeric Precursor with Controllable Carbon Structures for Highly Efficient Electromagnetic Wave Absorption at High Temperature,” *Carbon* 188 (2022): 12-24, https://doi.org/10.1016/j.carbon.2021.11.051.

8. J. Liu, Y. Feng, C. Liu, et al., “Novel SiBCN Composite Fibers with Broadband and Strong Electromagnetic Wave Absorption Performance,” *Journal of Alloys and Compounds* 912 (2022): 165190, https://doi.org/10.1016/j.jallcom.2022.165190.

9. J. Jiang, X. Deng, S. Li, X. Zeng, C. Wu, C. Yang, “Hierarchically Porous Multiphase Si-Based Ceramics with Synergistic Electromagnetic Wave Absorption Mechanisms,” *Advanced Science* 12 (2025): e10445, https://doi.org/10.1002/advs.202510445.

10. J. Liu, H. Li, H. Li, et al., “Deep-Sea Glass Sponges-Like Hollow Porous Ceramic Fiber Aerogel: Fabrication, Anti-Shrinkage and Thermal Insulation,” *Ceramics International* 50 (2024): 37714-37725, https://doi.org/10.1016/j.ceramint.2024.07.132.

11. W. Zou, X. Wang, Y. Wu, et al., “Opacifier Embedded and Fiber Reinforced Alumina-Based Aerogel Composites for Ultra-High Temperature Thermal Insulation,” *Ceramics International* 45 (2019): 644-650, https://doi.org/10.1016/j.ceramint.2018.09.223.

12. X. Liu, C. Su, Y. Zhong, X. Zhu, Z. Wu, S. Cui, “High Entropy (LaCeSmEuNd)_2_Zr_2_O_7_ Ceramic Aerogel with Low Thermal Conductivity and Excellent Structural Heat Resistance,” *Journal of the European Ceramic Society* 42 (2022): 5964-5972, https://doi.org/10.1016/j.jeurceramsoc.2022.06.075.

13. R. Zhang, Q. Qu, B. Han, B. Wang, “A Novel Silica Aerogel/Porous Y_2_SiO_5_ Ceramics with Low Thermal Conductivity and Enhanced Mechanical Properties Prepared by Freeze Casting and Impregnation,” *Materials Letters* 175 (2016): 219-222, https://doi.org/10.1016/j.matlet.2016.04.051.

14. F. He, Z. Zhu, L. Yang, et al., “Alumina Aerogels with Unidirectional Channels Under Different Freezing Temperatures During Freeze Casting——Part II: Anisotropic Mechanical and Thermal Conductive Properties,” *Ceramics International* 46 (2020): 25691-25696, https://doi.org/10.1016/j.ceramint.2020.07.045.

15. H. Li, Y. Chen, P. Wang, et al., “Porous Carbon-Bonded Carbon Fiber Composites Impregnated with SiO_2_-Al_2_O_3_ Aerogel with Enhanced Thermal Insulation and Mechanical Properties,” *Ceramics International* 44 (2018): 3484-3487, https://doi.org/10.1016/j.ceramint.2017.11.064.

16. Z. Xu, Y. Liu, Q. Xin, et al., “Ceramic Meta-Aerogel with Thermal Superinsulation up to 1700 °C Constructed by Self-Crosslinked Nanofibrous Network via Reaction Electrospinning,” *Advanced Materials* 36 (2024): 2401299, https://doi.org/10.1002/adma.202401299.

17. T. Huang, Y. Zhu, J. Zhu, H. Yu, Q. Zhang, M. Zhu, “Self-reinforcement of Light, Temperature-Resistant Silica Nanofibrous Aerogels with Tunable Mechanical Properties,” *Advanced Fiber Materials* 2 (2020): 338-347, https://doi.org/10.1007/s42765-020-00054-8.
